# Intermittent intravenous paracetamol versus continuous morphine in infants undergoing cardiothoracic surgery: a multi-center randomized controlled trial

**DOI:** 10.1186/s13054-024-04905-3

**Published:** 2024-04-30

**Authors:** Gerdien Zeilmaker-Roest, Christine de Vries-Rink, Joost van Rosmalen, Monique van Dijk, Saskia N. de Wildt, Catherijne A. J. Knibbe, Erik Koomen, Nicolaas J. G. Jansen, Martin C. J. Kneyber, Sofie Maebe, Greet Van den Berghe, Renata Haghedooren, Dirk Vlasselaers, Ad J. J. C. Bogers, Dick Tibboel, Enno D. Wildschut

**Affiliations:** 1 Department of Neonatal and Pediatric Intensive Care, Division of Pediatric Intensive Care, Erasmus MC-Sophia Children’s Hospital, Wytemaweg 80, 3015 CN Rotterdam, The Netherlands; 2 Department of Cardiothoracic Surgery, Erasmus MC, Rotterdam, The Netherlands; 3 Department of Biostatistics, Erasmus MC, Rotterdam, The Netherlands; 4 Department of Epidemiology, Erasmus MC, Rotterdam, The Netherlands; 5 Department of Pharmacology and Toxicology, Radboud University Medical Center, Nijmegen, The Netherlands; 6 Division of Systems Pharmacology and Pharmacy, Leiden Academic Centre for Drug Research, Leiden University, Leiden, The Netherlands; 7 Department of Clinical Pharmacy, St. Antonius Hospital Nieuwegein/Utrecht, Utrecht, The Netherlands; 8 Department of Pediatrics, Division of Pediatric Critical Care Medicine, Wilhelmina Children’s Hospital, University Medical Center Utrecht, Utrecht, The Netherlands; 9 Department of Pediatrics, Beatrix Children’s Hospital, University Medical Center Groningen, Groningen, The Netherlands; 10 Department of Pediatrics, Division of Pediatric Critical Care Medicine, Beatrix Children’s Hospital, University Medical Center Groningen, Groningen, The Netherlands; 11 Department of Intensive Care Medicine, UZ Leuven, Louvain, Belgium; 12 Department of Pediatrics, Maastricht University Medical Center+, MosaKids Children’s Hospital, Maastricht, The Netherlands

**Keywords:** Morphine, Intravenous paracetamol, Randomized controlled trial, Analgesia, Child, Congenital heart defects, Congenital cardiac surgery

## Abstract

**Background:**

To determine whether intermittent intravenous (IV) paracetamol as primary analgesic would significantly reduce morphine consumption in children aged 0–3 years after cardiac surgery with cardiopulmonary bypass.

**Methods:**

Multi-center, randomized, double-blinded, controlled trial in four level-3 Pediatric Intensive Care Units (PICU) in the Netherlands and Belgium. Inclusion period; March 2016–July 2020. Children aged 0–3 years, undergoing cardiac surgery with cardiopulmonary bypass were eligible. Patients were randomized to continuous morphine or intermittent IV paracetamol as primary analgesic after a loading dose of 100 mcg/kg morphine was administered at the end of surgery. Rescue morphine was given if numeric rating scale (NRS) pain scores exceeded predetermined cutoff values. Primary outcome was median weight-adjusted cumulative morphine dose in mcg/kg in the first 48 h postoperative. For the comparison of the primary outcome between groups, the nonparametric Van Elteren test with stratification by center was used. For comparison of the proportion of patients with one or more NRS pain scores of 4 and higher between the two groups, a non-inferiority analysis was performed using a non-inferiority margin of 20%.

**Results:**

In total, 828 were screened and finally 208 patients were included; parents of 315 patients did not give consent and 305 were excluded for various reasons. Fourteen of the enrolled 208 children were withdrawn from the study before start of study medication leaving 194 patients for final analysis. One hundred and two patients received intermittent IV paracetamol, 106 received continuous morphine. The median weight-adjusted cumulative morphine consumption in the first 48 h postoperative in the IV paracetamol group was 5 times lower (79%) than that in the morphine group (median, 145.0 (IQR, 115.0–432.5) mcg/kg vs 692.6 (IQR, 532.7–856.1) mcg/kg; *P* < *0.001*). The rescue morphine consumption was similar between the groups (p = 0.38). Non-inferiority of IV paracetamol administration in terms of NRS pain scores was proven; difference in proportion − 3.1% (95% CI − 16.6–10.3%).

**Conclusions:**

In children aged 0–3 years undergoing cardiac surgery, use of intermittent IV paracetamol reduces the median weight-adjusted cumulative morphine consumption in the first 48 h after surgery by 79% with equal pain relief showing equipoise for IV paracetamol as primary analgesic.

*Trial Registration* Clinicaltrials.gov, Identifier: NCT05853263; EudraCT Number: 2015-001835-20.

**Supplementary Information:**

The online version contains supplementary material available at 10.1186/s13054-024-04905-3.

## Background

Congenital heart disease is the most frequently diagnosed congenital defect, with a reported total prevalence of 8.0 in 1000 births in Europe [1] More than half of these patients require surgical intervention during the first 3 years of life.

An evidence-based guideline for pain treatment after cardiac surgery in children is lacking [2]. A 2022 guideline for pain and sedation management recommends opioids as primary analgesic in critically ill patients with moderate to severe pain [3].

A 2022 survey including over 200 European PICUs as well as a survey in pediatric cardiac ICUs showed large variation in dose and choice of analgesic drugs [4, 5]. Currently, most centers prescribe at least one opioid as primary analgesic after cardiac surgery [2].

Cardiopulmonary bypass (CPB) may alter pharmacokinetics (PK) after cardiac surgery [6]. Valkenburg et al. [7] showed that children after cardiac surgery with use of CPB have a lower clearance of morphine and a higher volume of distribution compared with non-cardiac surgery suggesting that children undergoing cardiac surgery with the use of CPB may need adjusted dosages of postoperative analgesics [8].

Opioids have been associated with potential serious adverse events, such as hypotension and respiratory depression and risk for opioid tolerance and opioid withdrawal [9]. These occurrences may result in prolonged ICU stay [10–13].

A possible alternative is paracetamol (acetaminophen), considered a safe drug when used in the age-appropriate dose [14, 15]. In an earlier randomized controlled trial, intermittent IV paracetamol proved equally effective as intravenous morphine in children up to one year of age after major non-cardiac surgery [16]. We therefore assumed that IV paracetamol might also benefit children after cardiac surgery.

## Methods

### Aims

We performed a prospective, multi-center, randomized double-blinded, controlled trial in children aged 0–3 years undergoing cardiac surgery with the use of CPB. The aim of the study was to test the hypothesis that intermittent IV paracetamol administration as primary analgesic after cardiac surgery will result in a reduction of at least 30% of the median weight-adjusted cumulative morphine dose (in mcg/kg) during the first 48 h after cardiac surgery.

### Study design and setting

A prospective, multi-center, randomized double-blinded controlled trial conducted in four level-3 PICUs in the Netherlands and Belgium (Erasmus MC-Sophia Rotterdam, Wilhelmina Children’s Hospital UMC Utrecht, Beatrix Children’s Hospital UMC Groningen, the Netherlands and University Hospital Leuven, Belgium).

The study was approved by the Erasmus MC Medical Ethics Committee and was registered in the Dutch trial registry under the code NTR5448 as well at the Central Committee on Research Involving Human Subjects (NL 53085.078.15), EudraCT (2015-001835-20-NL/BE), and clinicaltrials.gov (identifier: NCT05853263).

Parents or legal guardians of the participating children provided written informed consent prior to any study procedures. The study protocol is published in *Trials* and available online [17].

### Patients

#### Inclusion criteria

Children (0–36 months) admitted to the PICU after cardiac surgery with the use of CPB between March 2016 and July 2020 were eligible to participate.

#### Exclusion criteria

No informed consent, a known allergy or intolerance for paracetamol or morphine, opioids administered in the 24 h before surgery, hepatic dysfunction prior to surgery (defined as three times the reference value of alanine/aspartate aminotransferase (ALAT/ASAT)), and/or renal insufficiency defined at least as RIFLE category Risk prior to surgery.

Withdrawal criteria:

- Withdrawn informed consent
- Signs of hypersensitivity or an allergic reaction to either morphine or paracetamol
- Re-operation or extracorporeal membrane oxygenation (ECMO) treatment within 48 h
- Hepatic dysfunction, defined as three times the reference value of ALAT/ASAT
- Renal insufficiency defined as RIFLE category Injury
- Administration of muscle relaxants after surgery for 3 h or longer
- Body temperature of 38.5° Celsius after surgery for 6 h or longer

### Randomization, blinding and treatment allocation

Blocked randomization with randomly chosen block sizes and stratification by center was applied. A biostatistician (JvR) carried out the randomization in advance, with a randomization schedule for each participating center. Participants were assigned a consecutive trial number on the randomization schedule. The randomization schedule was safely stored in the local pharmacy at every center. The hospital pharmacies of all participating centers, but not the physicians, had access to the randomization schedule to ensure concealed allocation. Study medication was prepared at the participating centers by the pharmacy. In case of a medical emergency the pharmacists could be consulted on treatment allocation. To ensure blinding in both groups, a double dummy (intermittent placebo bolus of Sodium chloride (NaCl) 0.9% in a similar volume as the IV paracetamol dose or a continuous placebo infusion of NaCl 0.9% at the same rate as an equivalent morphine continuous infusion) was used.

### Procedures

#### Peroperative management

Peroperative analgosedation in each center was performed per local protocol. Only short acting opioids were used during surgery*.* Type of analgesics and sedatives were registered in the electronic patient data management system, but not included in the case record form (CRF). Only short acting opioids were used during surgery. Information on the duration of surgery, type of CPB system used, CPB run time, aorta cross clamp time and degree and duration of hypothermia were registered in the CRF.

### Assessments

Trained pediatric ICU nurses applied the Numeric Rating Scale-11 (NRS-11) pain and COMFORT-Behavior Scale (COMFORT-B) every 2 h. Both instruments are validated in critically ill children [18].

Pediatric delirium was assessed thrice daily in children requiring sedatives or analgesics for more than 48 h using the validated SOS-Pediatric Delirium (SOS-PD) scale [19].

All patients were classified according to the Risk-Adjusted Classification for Congenital Heart Surgery (RACHS-1) score [20]. The Pediatric Risk of Mortality (PRISM) lll score [21], combined with the Pediatric Logistic Organ Dysfunction 2 (PELOD-2) [22] were assessed on the day of surgery postoperatively, and postoperative days 1 and 2. To evaluate inotropic support in the first 48 h after surgery we calculated the vasoactive inotropic score (VIS) using the highest recorded inotropic and vasopressor doses. VIS was calculated as follows: *dobutamine dose (mg/kg/min)* + *dopamine dose (mg/kg/min)* + norepinephrine dose (mg/kg/min) *100 + epinephrine dose (mg/kg/min) *100 + milrinone dose (mg/kg/min) *10 + vasopressin dose (U/kg/min) *10,000 [23, 24].

To assess renal injury the lowest glomerular filtration rate (GFR) in the first 48 h after surgery was recorded and categorized using the KDIGO criteria [25].

### Study protocol

A loading dose of morphine 100 mcg/kg IV was administered to all patients after separation from CPB, directly after surgery. Hereafter, patients were randomized to receive either continuous morphine or intermittent IV paracetamol. Paracetamol was dosed according to the Dutch Pediatric Formulary (loading dosage 20 mg/kg in all patients, maintenance dosage 40 mg/kg/day in neonates and 60 mg/kg/day in all other patients, 4 times daily) [26]. Morphine dosing was based on a population PK model-derived dosing regimen, range 3.9 to 16.0 mcg/kg per hour, resulting in similar morphine concentration across children’s age and bodyweight ranges [9, 17, 27, 28].

Pain or discomfort was scored with both the NRS pain scale and COMFORT-B. An open label morphine IV rescue dose (10 mcg/kg in neonates < 10 days, 15 mcg/kg in older patients) was administered if NRS-score ≥ 4. Pain was re-evaluated 10 min after the intervention. If pain persisted after three rescue doses, a morphine loading dose of 100 mcg/kg was administered and open label continuous morphine infusion was started at 10 mcg/kg per hour. Open label morphine infusion could be increased to maximum 30 mcg/kg per hour. In case of inadequate analgesia with maximum open label morphine, patients were switched to continuous IV fentanyl. Open label morphine or fentanyl infusion was titrated to effect using the NRS pain scale. In each group, continuous study morphine infusion was decreased on postoperative day 1 if the NRS score < 3 and COMFORT-B score < 10. Study medication was continued until 48 h after surgery. In patients who met the withdrawal criteria, treatment allocation was de-blinded and trial morphine infusions and/or paracetamol were switched to equivalent open label dose infusions of both paracetamol and morphine and analyzed as intention to treat within their treatment arm.

### Outcomes

The primary endpoint is median weight-adjusted cumulative morphine dose in mcg/kg during the first 48 h postoperatively.

Secondary outcomes are:

1. Morphine rescue dose in micrograms per kilogram in the first 48 h postoperatively, number of patients receiving rescue morphine doses, and number of patients needing rescue morphine continuous infusions.
2. Incidence of adverse drug reactions:
  - Hemodynamic; hypotension or bradycardia, with the need for medication or a fluid bolus
  - Decreased gastrointestinal motility or intestinal obstruction not directly related to the underlying diagnosis and not previously existing obstruction with the need for intervention
  - Vomiting
  - Number of reintubations
  - Pediatric delirium (SOS-PD > 3)
3. Non-inferiority analysis by comparing the proportion of patients with one or more NRS scores of at least 4 between study arms
4. Average COMFORT-B score
5. Concomitant use of sedatives (type and dose)
6. Number of hours on mechanical ventilation
7. Length of PICU stay

Secondary outcomes were registered until 48 h after stop trial medication (96 h after surgery).

### Statistical methods

#### Power analysis

The power analysis, conducted in a simulation study, is based on a comparison of the primary outcome between groups using a Mann–Whitney test. For the simulation study, data from a previous study were used [16]. Based on this data set, the median weight-adjusted cumulative morphine consumption in the control group would be 357 mcg/kg (IQR: 220–605), and was hypothesized that this morphine consumption would be reduced by 30% in the intervention (paracetamol) group. The simulation study showed that using a two-sided significance level of 5%, 86 patients per group would be required to obtain a power of 95%.

In the study design phase we already anticipated some patient exclusions and missing data. Therefore the recruitment target was set with some margin for error to 104 patients per group (208 in total), to compensate for the effects of patient exclusions/missing data as well as the statistical adjustment for stratification by center.

### Statistical analysis

For the comparison of the primary outcome between groups, the nonparametric Van Elteren test with stratification by center was used. Linear regression analysis with group and treatment center as categorical predictor variables was applied for the secondary outcomes.

For comparison of the proportion of patients with one or more NRS scores of 4 and higher between the two groups, a non-inferiority analysis was done, using a non-inferiority margin of 20%. The confidence interval was calculated with the method of Klingenberg[29], with adjustment for center. Adverse effects were compared between groups using Fisher exact tests. The level of significance was set to 5%, and all tests were two-sided.

### Interim analysis and stopping guidelines

An international external Data and Safety Monitoring Board (DSMB) composed of an experienced cardiac surgeon, pediatric intensivist and cardio-anesthesiologist together with an independent biostatistician was installed. The study protocol did not contain an interim analysis. The DSMB evaluated inclusion rate and safety of participants (need for rescue morphine and NRS pain scores in both groups) 4 times during the inclusion period and advised us to continue the study without design changes to the protocol. To better assess safety aspects and before patient enrolment, morphine rescue dose in micrograms per kilogram in the first 48 h postoperatively, number of patients receiving rescue morphine doses, number patients needing rescue morphine continuous infusions and average COMFORT-B scores were added as secondary outcome.

## Results

### Patient characteristics

**Fig. 1:**
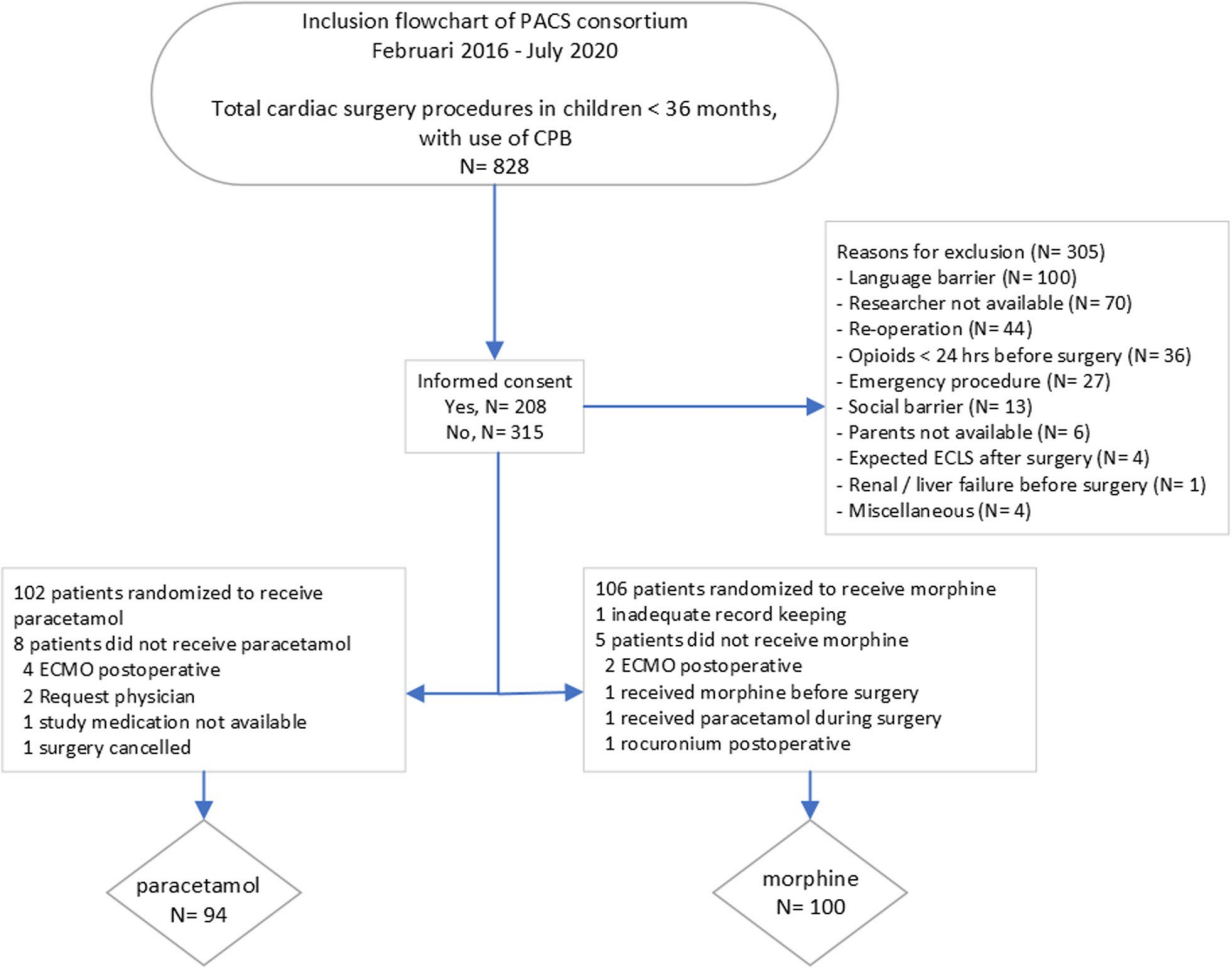
Study flowchart

Fourteen of the enrolled 208 children were withdrawn from the study before start of study medication leaving 194 patients for final analysis. The main reasons are noted in the flowchart in Fig. 1.

The morphine group contained 100 patients versus 94 in the intravenous paracetamol group. The two groups did not significantly differ in patient characteristics and risk of mortality scores (Table 1. Patient characteristics). Underlying cardiac diagnoses are shown in Table 2.

**Table 1:** Patient characteristics

Characteristic	Paracetamol (n, %) N = 94	Morphine (n, %) N = 100
Sex, n (%) Male Female	51 (54) 43 (46)	52 (52) 48 (48)
Center, n (%) Erasmus MC-Sophia Rotterdam Wilhelmina Children’s Hospital UMC Utrecht Beatrix Children’s Hospital UMC Groningen University Hospital Leuven	59 (62.8) 14 (14.9) 7 (7.4) 14 (14.9)	61 (61) 14 (14) 8 (8) 17 (17)
Age at surgery in months, median (IQR)	5 (3–10)	4 (2–7)
Body weight in kg, median (IQR)	5.8 (4.8–8.6)	5.9 (4.5–7.5)
Aortic Clamp Time, minutes, median(IQR)	58.0 (30.5–82.5)	61.5 (46.0–82.8)
Survival	92 (98%)	98( 98%)
CPB duration in min, median (IQR)	98.0 (60–138)	98.5 (79–133)
RACHS, n (%) 1 2 3 4 5 6	12 52 22 5 – 3	4 62 20 15 – 0
PRISM III, median (IQR)	15 (9)	16 (9)
PIM II, median(IQR)	− 3.78 (0.58)	− 3.73 (0.51)
PELOD		
Day 0 Day 1 Day 2	5 (3) 4 (4) 2 (5)	5 (3) 3 (4) 2 (4)

UMC = University medical center, IQR = interquartile range, CPB = cardiopulmonary bypass, PRISM III = Pediatric risk of Mortality-lll score, PIM II = Pediatric Index of Mortality, RACHS = Risk Adjusted Classification for Congenital Heart Surgery (RACHS-1) score, NRS = Numeric Rating Scale VIS = Vasoactive Inotropic Score PICU = Pediatric Intensive Care Unit

**Table 2:** Underlying cardiac diagnosis per group

Intermittent IV paracetamol (n = 94)	Number (%)
VSD	20 (21.3)
TOF	16 (17)
AVSD	15 (16)
ASD	12 (12.7)
TGA	7 (7.4)
Valve stenosis/insufficiency	6 (6.3)
HRHS	5 (5.3)
HLHS	4 (4.3)
PAPVR	3 (3.2)
Hypoplastic aortic arch	2 (2.1)
TAPVR	2 (2.1)
Other *	2 (2.1)
Morphine (n = 100)	
VSD	29 (29)
TOF	21 (21)
AVSD	12 (12)
TGA	10 (10)
Hypoplastic aortic arch	5 (5)
HLHS	8 (8)
Valve stenosis/insufficientie	4 (4)
ASD	2 (2)
HRHS	2 (2)
PAPVR	3 (3)
VSD & ASD	2 (2)
Other **	2 (2)

*Other: ALCAPA, Ebstein with pulmonary valve atresia

**Other: Truncus arteriosus, TAPVR

Abbreviations: VSD: ventricular septal defect, TOF: Tetralogy of Fallot, AVSD: atrioventricular septal defect, ASD: atrial septal defect, TGA: transposition of the Great Arteries, HRHS: hypoplastic right heart syndrome, HLHS: hypoplastic left heart syndrome, TAPVR: total abnormal pulmonary venous return, PAPVR: partial abnormal pulmonary venous return, ALCAPA (anomalous left coronary artery from the pulmonary artery)

Twenty-eight patients in the IV paracetamol group and 26 patients in the continuous morphine group were switched to open label IV PCM and continuous morphine during the study period, most often because of fever. Reasons for withdrawal and time to withdrawal are shown in Table 3. All patients were analyzed in their primary allocated group using the Fisher exact test.

**Table 3:** Time and reasons for patient withdrawal

Endpoint	Paracetamol (n = 28)	Morphine (n = 26)	
Time to withdrawal (hr:min) (Median(IQR)	16:00 (11:29–19:23)	15:36 (9:03–18:48)	0.67
Reasons for withdrawal, n (%)			
Fever	13 (46.4)	12 (46.2)	0.815
Parental request	2 (7.1)	3 (11.5)	
Physicians request	4 (14.3)	6 (23.1)	
Trial medication delivery problem	4 (14.3)	4 (15.4)	
ECMO/OR/muscle relaxants	5 (17.9)	1 (3.8)	

ECMO: extracorporeal membrane oxygenation, OR: operation

### Study outcomes

The median weight-adjusted cumulative morphine dose in the first 48 h postoperative in the paracetamol group was 5 times lower (79%) than in the morphine group (*P* < *0.001* (Table 4)).

**Table 4:** Primary and secondary study outcomes

Outcome	Paracetamol (n = 94)	Morphine (n = 100)	P-value
Cumulative morphine consumption, (Median (IQR)), mcg/kg*	145.0 (115.0–432.5)	692.6 (532.7- 856.1)	< 0.001
Rescue morphine dose total, median (IQR), mcg/kg*	29.4 (0–45.7)	30.0 (0–70.9)	0.38
Patients with rescue morphine bolus, (n) (%)*	62 (66.0%)	69 (69.0%)	0.76
Patients with rescue morphine infusions (n)*	40(42.3%)	42 (42.0%)	1.00
Co-medication (n)*			
Midazolam Lorazepam Propofol Fentanyl Clonidine Ketamine Dexmedetomidine	76 (80.9%) 5 (5.3%) 26 (27.7%) 8 (8.5%) 6 (6.4%) 23 (24.5%) 6 (6.4%)	77 (77%) 10 (10%) 19 (19%) 10 (10%) 5 (5%) 21 (21.0%) 8 (8%)	0.59 0.29 0.18 0.81 0.67 0.54 0.68
PICU stay in days ( median(IQR))	3 (2–6)	2 (1–5)	0.10
Duration postoperative mechanical ventilation (median, IQR, hours)	11.8 (4.5–45.6)	15.4 (5.7–28.8)	0.39
Reintubation (n, %)*	3 (3.2)	2 (2)	0.68
Adverse events**			
Hemodynamic instability (bradycardia or hypotension) Gastrointestinal (obstruction, obstipation, vomiting) Delirium Apnea	22 (23.4%) 3 (3.2%) 3 (3.2%) 3 (3.2%)	28 (28%) 1 (1%) 5 (5%) 2 (5%)	0.46 0.28 0.53 0.60
VIS	5 (0–9.6)	5 (3.0–10)	0.21
NRS ≥ 4 at least once (n,%)*	55 (59%)	62 (62%)	0.62
Comfort-B scale scores (median, range)*	12 (8–22)	12 (7–20)	0.05

*48 h postoperative

** 96 h postoperative

PICU = Pediatric Intensive Care Unit, NRS = Numeric Rating Scale, VIS = Vasoactive Inotropic Score

There were no significant differences between groups in median total rescue morphine consumption in the first 48 h postoperative, proportion of patients receiving rescue morphine boluses and additional continuous morphine infusions.

The median weight-adjusted cumulative morphine consumption in the 194 patients who received the study medication for 48 h was 145.0 (115.0–432.5) mcg/kg in the IV paracetamol group vs 692.6 (532.7–856.1) mcg/kg (p < 0.001) in the continuous morphine group. The rescue morphine consumption was similar in both groups, 29.4 (0–45.7) mcg/kg in the IV paracetamol group vs 30.0 (0–70.9) mcg/kg in the continuous morphine group. The percentage of patients needing continuous open label morphine was 42.3% in the IV paracetamol group vs 42% in the continuous morphine group.

Percentage of adverse effects did not significantly differ between treatment groups (Table 4). Hemodynamic instability as predefined was the most frequently occurring adverse effect in both groups; 22 (23%) in the IV paracetamol group versus 28 (28%) in the continuous morphine group (p = 0.46). Inotropic support expressed as the vasoactive inotropic score (VIS) based on the highest doses of inotropes in the first 48 h was similar between both groups (Table 4) (See Additional file 2, Table 1: Inotropic support in the first 48 h). 

Three patients in IV paracetamol group were re-intubated versus two patients in the continuous morphine group (p = 0.68).

Overall median NRS pain scores were 0 (IQR 0–3) vs 0 (IQR 0–5) in the IV paracetamol and continuous morphine group, respectively. The number of patients with one or more NRS pain scores of 4 or higher was similar in both groups (Table 4).

The estimate (two-sided 95% CI) for the difference in proportions between the paracetamol group and the continuous morphine group, adjusted for center, was − 3.1% (95% CI − 16.6%–10.3%). Non-inferiority of IV paracetamol administration was proven because the upper bound of the 95% CI was below the predefined non-inferiority margin of 20%.

The concomitant use of sedatives was similar in both groups (Table 4). An overview of the doses given of the used sedatives during the study period are displayed in the Additional file 1 (Table 3: Sedative use in the first 48 h).

Nine patients had hepatic dysfunction (ALAT n = 3 (morphine n = 2, paracetamol n = 1); ASAT n = 8 (morphine n = 4, paracetamol n = 4). Renal dysfunction ranging from mild to severe ( GFR under 60 ml/min) was seen in 48 patients (Table 5). Severe renal dysfunction defined as a GFR of 29 ml/min or less was seen in 8 patients; 6 in the morphine group and 2 in the IV Paracetamol group. Most of these patients (31 vs 17) received study morphine, this was not significantly different between groups (p = 0.71 (2-sided)). There seems to be a trend toward increased renal dysfunction in the morphine group which might support reports of protective aspects of IV paracetamol in these patients.

**Table 5:** Renal dysfunction in GFR per study group

Renal dysfunction	Morphine	IV Paracetamol	P-value
*GFR 45–59 ml/min (mild to moderate dysfunction, n)*	13	8	
*GFR 30–44 ml/min (moderate to severe dysfunction)*	11	8	0.71
*GFR 15–29 ml/min (severe dysfunction)*	6	2	
**Hepatic dysfunction**			
ALAT	2	1	0.60
ASAT	4	4	0.93

GFR: Glomerular Filtration Rate, ALAT: alanine aminotransferase, ASAT: aspartaat aminotransferase.

## Discussion

In this multi-center RCT, infants below 4 years of age treated with IV paracetamol as primary analgesic after cardiac surgery with CPB received significantly less morphine within the first 48 h after surgery than did morphine. Their median weight-adjusted morphine dose was 79% lower than that of patients receiving continuous morphine as a primary analgesic.

Several studies have shown opioid-sparing effects of intravenous or rectal paracetamol in children of various ages undergoing various types of non-cardiac surgery [16, 30–33]. While these earlier studies showed a 15–66% reduction in total morphine consumption, this reduction in total morphine dosing in our cohort was higher at 79%. Differences in study populations and study designs may prevent a true comparison of the results. Whether altered PK or PD in our patient cohort plays a role in our findings is unclear but our findings are similar to our earlier study in non-cardiac patients suggesting that CPB does not greatly influence PK or PD parameters [16].

Adding other analgesics such as NSAIDs or alpha2 antagonists such as dexmedetomidine could potentially reduce morphine requirements. Adults studies have shown a reduced opioid consumption when using dexmedetomidine. In children, favorable effects of dexmedetomidine to reduce opioid consumption or pain scores have not been proven [9].

Although data is sparse both in efficacy as well as in safety in this patient group there is some evidence that NSAID’s could potentially reduce morphine use [34–36].

Morphine has both sedative and analgesic properties. Interestingly, approximately 80% of our included patients in either group needed additional sedatives although the use of additional sedatives was similar in both groups. This proportion is higher than that in a similar study by Ceelie et al. in non-cardiac surgery patients, in which between 7.9 and 15.2% of patients received additional midazolam [16] and in a similar study by de Hoogd et al., in which 37.2% of all patients received additional continuous midazolam [37] The discrepancy may be explained by the difference in patient ages between the studies: median five months in our study vs. less than one month in the study by Ceelie et al. [16] Older children may need more sedatives to accept intubation and mechanical ventilation, chest tubes and the hospital environment compared to neonates. De Hoogd et al. used much higher morphine doses compared to our study; leading to lower use of other sedatives as morphine itself has a sedative effect as well.

Lower or even absent continuous morphine infusions might therefore lead to higher needs for sedatives. We found no difference in midazolam usage or cumulative midazolam doses (Additional file 1: Additional Table 3. Sedative use in the first 48 h) between the two study groups, suggesting that sedative needs were not influenced by the morphine infusion. Furthermore, several studies report similar use of midazolam in children following cardiac surgery treated with continuous morphine infusions as found in our study [38–40].

The most common adverse effects of the study drugs were hemodynamic instability which was comparable between groups; 23.4% vs 28.0% for IV paracetamol and continuous morphine, respectively. Moreover inotropic support expressed as the VIS score in the first 48 h was not significantly different between groups. Changes in hemodynamic parameters are multifactorial and differentiation between morphine effect or purely cardiac-surgery-related hemodynamic effects is difficult. Adverse effects of IV paracetamol also need to be considered. In a previous study, IV paracetamol was associated with hypotension in 5% of children in the cardiac intensive care unit. This can partially explain why there is no difference in hemodynamic instability between both study groups. Ultimately we found no differences in complications but could not establish a correlation between medication doses and adverse effect in a one on one relationship.

Another potential complication in our patients is renal or hepatic failure. Data on acute kidney injury associated with paracetamol are somewhat ambiguous. Renal failure has been reported in patients with a paracetamol overdose [41]. On the other hand there is some evidence that suggests that paracetamol protects against kidney injury mediated by free hemoglobin in both animals and humans. Xiong et al. suggest that early postoperative paracetamol may even be beneficial in preventing acute kidney injury [42]. Although not statistically different the patients in the morphine group did have a higher incidence of kidney injury compared to the IV paracetamol group (31% vs 18%) (p0.71). These findings might support the earlier publications on the protective aspects of PCM in these patients. Hepatic failure was very limited in our patients. Renal and hepatic dysfunction recovered after discontinuation of the study medication.

Reintubation was rare in both groups and could not be attributed to pharmacologic induced hypopnea or apnea. Length of PICU stay as well as time on mechanical ventilation did not differ between groups: This suggests no adverse respiratory effects of the higher morphine exposure in the continuous morphine group. Gastrointestinal side effects as well as withdrawal syndrome or delirium are side effects that might be more prominent after 96 h after surgery. Since all patients switched to open label morphine and paracetamol after 48 h, late onset adverse effects could have been masked or missed due to the short follow-up time.

### Strengths

To the best of our knowledge, this is the first large randomized controlled trial in children under the age of 4 years undergoing cardiac surgery with CPB, and the first to compare IV paracetamol as a primary analgesic postoperatively with the common practice of administering opioids.

### Limitations

About 30% of patients in either group were switched to open label paracetamol and morphine during the study timeframe. In approximately half of these cases the reason was sustained fever after surgery and the need for IV paracetamol to decrease body temperature. Interestingly, fever episodes were similar in both groups. Changing to open label might have actually increased the morphine consumption in patients in the IV paracetamol group who were switched from placebo to open label morphine within 48 h, thereby reducing the observed difference between the groups. The need for open label paracetamol due to fever does not reflect inadequate pain management and would not be an issue in open label paracetamol treatment.

To conclude, administration of intermittent intravenous paracetamol as primary analgesic in children under 3 years of age after cardiac surgery with the use of cardiopulmonary bypass resulted in a substantial reduction of the median weight-adjusted cumulative morphine consumption in the first 48 h postoperatively and reduced the need for continuous morphine infusions in almost 60% of all patients treated with IV paracetamol. Considering the similar need for rescue morphine doses and the same median NRS and COMFORT-B scores in both groups, an analgesic treatment protocol incorporating a loading dose of 100 mcg/kg morphine followed by IV paracetamol maintenance doses and bolus rescue morphine achieves equally effective postoperative pain relief in these patients independent of diagnosis or type of cardiac surgery negating the need for continuous morphine infusions in 40% of all patients.

### Supplementary Information

**Additional file 1.** **Additionale Table 3**. Sedative use in the first 48 h.

**Additional file 2.** **Additional Table 1**. Inotropic support in the first 48 h.

## Data Availability

The datasets used and/or analyzed during the current study are available from the corresponding author on reasonable request.

## Abbreviations

IV: Intravenous
(P)ICU: (Pediatric) Intensive care Units
NRS: Numeric Rating Scale
CPB: Cardiopulmonary Bypass
PK: Pharmacokinetics
ALAT: Alanine aminotransferase
ASAT: Aspartate aminotransferase
ECMO: Extracorporeal Membrane Oxygenation
NaCL: Sodium Chloride
CRF: Case record form
COMFORT B: COMFORT Behavior Scale
SOS-PD: SOS-Pediatric Delirium
RACHS-1: Risk-Adjusted Classification for Congenital Heart Surgery
PRISM III: The Pediatric Risk of Mortality III
PELOD2: Pediatric Logistic Organ Dysfunction 2
DSMB: Data and Safety Monitoring Board
