# Supplementary material for: Intermittent intravenous paracetamol versus continuous morphine in infants undergoing cardiothoracic surgery: a multi-center randomized controlled trial
Source: Crit Care. 2024 Apr 30;28:143. doi: 10.1186/s13054-024-04905-3 (PMC11061924; doi:10.1186/s13054-024-04905-3)
Supplement: Supplementary file 2 — Additional file 2. Additional Table 1. Inotropic support in the first 48 h. [file 13054_2024_4905_MOESM2_ESM.pdf]

# PACS STUDY:

# Pediatric Analgesia after Cardiac Surgery

**Morphine IV versus paracetamol IV after cardiac surgery in neonates and infants.**

**(Juny 2018, Version 4)**

| | |
|---|---|
| **Protocol ID** | **NL 53085.078.15** |
| **Short title** | **Morphine IV vs Paracetamol IV for analgosedation in neonates and infants after cardiac surgery** |
| **EudraCT number** | ***2015-001835-20*** |
| **Version** | **4.0** |
| **Date** | **14-06-2018** |
| **Coordinating investigator/project leader** | ***Dr. E.D. Wildschut, Erasmus MC***<br>***Prof. Dr. M. van Dijk, Erasmus MC*** |
| **Principal investigator(s) (in Dutch: hoofdonderzoeker/ uitvoerder)**<br>***<Multicenter research: per site>*** | ***Dr. E. Wildschut, Erasmus MC***<br>***Dr. M.C.J. Kneyber, UMCG***<br>***Dr. N.J.G. Jansen, Wilhelmina Children's Hospital UMCUtrecht***<br>***Drs. E. Koomen, Wilhelmina Children's Hospital UMCUtrecht***<br>***Prof. dr. D. Vlasselaers, UZ Leuven***<br>***Prof. dr. G. van den Berghe, UZ Leuven***<br>***Dr. S. Maebe, UZ Leuven*** |
| **Sponsor (in Dutch: verrichter/opdrachtgever)** | ***Erasmus MC, Rotterdam*** |
| **Subsidising party** | ***ZonMw, goed gebruik geneesmiddelen programma*** |

| | |
|---|---|
| **Independent expert (s)** | **Dr. H. IJsselstijn, Erasmus MC**<br>**Dr. T.F.W. Wolfs, Wilhelmina Children's Hospital UMCUtrecht**<br>**Drs. R.G.T. Blokpoel, UMCG**<br>**Prof. dr. B. Eyskens, UZ Leuven** |
| **Laboratory sites <*if applicable*>** | ***Erasmus MC, Rotterdam*** |
| **Pharmacy <*if applicable*>** | ***Erasmus MC, Rotterdam*** |

| | | | |
|---|---|---|---|
| **Amendment 1** | **Administrative** | **19-04-2016** | **Replacement of DSMB member prof.dr. K. Allegaert by dr. C. Breatnach** |
| **Amendment 3** | **Administrative** | **10-07-2017** | - **Adjustments to SAE reporting for this study.**<br>- **Laboratory measurement will not be done only for study purposes.**<br>- **Addition to PK sampling times.**<br>- **Removal of LUMC and addition of Leuven UZ as participating centre.** |
| **Amendment 4** | **Administrative and substantial** | | - **postponing end date of study**<br>- **adding stress hormone measurements at Erasmus MC-Sophia children's hospital and UZ Leuven** |

**PROTOCOL SIGNATURE SHEET**

| **Name** | **Signature** | **Date** |
|---|---|---|
| **Sponsor or legal representative:**<br><br>***Prof. dr. D. Tibboel***<br><br>**Head of Department:**<br><br>***Prof. dr. R.H.M. Wijnen***<br><br>**Principal Investigators & Project leader:**<br><br>***Dr. E.D. Wildschut, Erasmus MC***<br><br>***Prof. Dr. M. van Dijk, Erasmus MC*** | | |

TABLE OF CONTENTS


1. Introduction and rationale ..... 9
2. Objectives ..... 18
3. Study design ..... 19
4. Study population ..... 20
4.1 Patients ..... 20
4.2 Inclusion criteria ..... 20
4.3 Exclusion criteria ..... 20
4.4 Sample size calculation ..... 20
5. Treatment of subjects ..... 22
5.1 Investigational product/treatment ..... 22
5.2 Rescue analgesic medication ..... 22
6.1 Main study parameter/endpoint ..... 24
6.1.1 Secondary study parameters/endpoints (if applicable) ..... 24
6.1.2 PD outcome measurements ..... 24
6.1.3 PK analysis and Blood sampling regimen: ..... 27
6.2 Randomization, blinding and treatment allocation ..... 28
6.3 Study procedures ..... 30
6.4 Withdrawal of individual subjects ..... 31
6.4.1 Specific criteria for withdrawal ..... 31
6.5 Replacement of individual subjects after withdrawal ..... 32
6.6 Follow-up of subjects withdrawn from treatment ..... 32
6.7 Premature termination of the study ..... 32
7. Safety reporting ..... 33
7.1 Section 10 WMO event ..... 33
7.2 AEs and SAEs ..... 33
7.2.1 Adverse events (AEs) ..... 33
7.2.2 Serious adverse events (SAEs) ..... 33
7.3 Follow-up of adverse events ..... 34
7.4 Data Safety Monitoring Board (DSMB) ..... 34
8. Statistical analysis ..... 36
9. Ethical considerations ..... 37
9.1 Regulation statement ..... 37
9.2 Recruitment and consent ..... 37
9.3 Objection by minors or incapacitated subjects (if applicable) ..... 37
9.4 Benefit and risks assessment, group relatedness ..... 37
9.5 Compensation for injury ..... 38
10. Administrative aspects and publication ..... 39
10.1 Handling and storage of data and documents ..... 39

10.2 Monitoring and Quality Assurance ........ 39
10.3 Amendments ........ 39
10.4 Annual progress report ........ 39
11. References ........ 40
Appendix 1: summary of results worldwide survey ........ 46
Appendix 2: morphine dosing regimen ........ 47
Appendix 3: sedation protocol (page 1) ........ 48
Appendix 4: post-operative pain protocol in children 0-3 years of age ........ 52
Appendix 5: Comfort assessment scale. ........ 53
Appendix 6: Visual Analogue Score ........ 54
Appendix 7: Parents Postoperative Pain Measure-Short Form ........ 55
Appendix 8: Sophia Ontwenningsverschijnselen Scorelijst-Pediatrisch Delirium scale ........ 56
Appendix 9: anaesthetic management ........ 57
Appendix 10: monitoring plan ........ 58

**Summary**

**Rationale:**

Post-operative pain relief after cardiac surgery in children is mainly achieved by opioids. However, worldwide dosing and choice of opioids used vary greatly, with morphine being the drug of first choice. Morphine has unwanted hemodynamically and respiratory side effects. Post cardiac surgery patients may therefore potentially benefit from a non-opioid drug for their pain relief. A previous study has shown that intravenous paracetamol is effective and opioid sparing in children after major non cardiac surgery. Intermittent intravenous administration of paracetamol may therefore result in a significant decrease in cumulative morphine consumption in the first 48 hours after cardiac surgery.

**Objective:**

The aim of the study is to test the hypothesis that intermittent intravenous paracetamol administration in children after cardiac surgery will result in a reduction of at least 30% of the cumulative morphine requirement.

**Study design:**

A prospective multi center  randomized double blind study.

**Study population:**

Children younger than four years of age, admitted to the pediatric ICU's of Erasmus MC- Sophia Rotterdam, Wilhelmina Children's Hospital UMC Utrecht, Beatrix children's hospital Groningen or Leuven University Hospital,  after cardiac surgery.

**Intervention:**

Patients will be randomized to receive either intermittent intravenous paracetamol or continuous intravenous morphine up to 48 hours post-operatively. Morphine will be available as rescue medication for both groups.

**Main study parameters/endpoints:**

The cumulative morphine consumption during the first 48 hours after cardiac surgery.

**Nature and extent of the burden and risks associated with participation, benefit and group relatedness:**

Possible burden and risk of participating in the study is the risk of insufficient analgesia after cardiac surgery with intermittent intravenous paracetamol. Given previous studies on the postoperative efficacy of paracetamol in children after major non cardiac surgery, this risk is considered low. Moreover, morphine will be administered as rescue medication in case of insufficient analgesia in both groups.

Paracetamol in therapeutic doses has been widely studied in children and is considered to be efficacious and safe in the proposed study population. In addition, study participants may experience less morphine-related side effects if paracetamol reduces the total morphine requirement.

The study is essential in this population as results from adults or healthy children cannot be extrapolated to the delicate situation in postoperative cardiac surgery children. Differences in age, underlying (cardiac) disease, and use of cardiopulmonary bypass will result in different pharmacokinetics and pharmacodynamics of both paracetamol and morphine after cardiovascular surgery in children.

## 1. Introduction and rationale

Congenital heart disease (CHD) is the largest group of congenital defects, accounting for almost one-third of all congenital anomalies (1). In Europe, the reported total prevalence of CHD is 8.0 in 1000 births, with some variability between countries (2). In the Netherlands approximately 800 children with congenital cardiac defects undergo cardiac surgery every year in one of the four designated cardiac surgery centers (Rotterdam, Leiden, Utrecht, Groningen). Surgical intervention is necessary within the first year of life in 55% and in 67% during the first three years of life (3).

After the publication by Anand and Aynsley-Green (4) the importance of adequate postsurgical pain relief in neonates and infants became apparent. Findings that untreated pain results in prolonged behavioral consequences and increases stress hormone levels have resulted in an increased use of morphine in neonates and children for post-operative pain relief (5-8). Continuous IV morphine has been shown to be effective for post-operative pain relief in over 200 post-operative non cardiac surgery patients between 0 and 3 years of age with a clear age difference in morphine requirements between neonates and older patients (9).

Gestational and postnatal age, genetic background, organ maturation, and critical illness all influence PK resulting in differences in plasma concentrations of drugs (10-13) . In 2009 Knibbe et al. proposed a novel morphine dosing regimen for neonates and children based on extensive PK studies resulting in a significant dose reduction of morphine in neonates < 10 days post-natal age (10). This model was further validated with new data sets (14) and ultimately resulted in a prospective randomized controlled trial in which the proposed dosing regimen for morphine dosing was evaluated (15). While the model accurately predicted morphine and metabolite concentrations throughout the population studied, it showed that older children needed additional morphine doses, potentially because of reduced sensitivity to morphine compared to neonates (15). Finally IV paracetamol was compared to morphine as a primary analgesic drug in a recent randomized controlled trial in postoperative children. Ceelie et al (16) showed effective pain relief of intravenous paracetamol and IV morphine after major non-cardiac surgery in neonates and infants up to 1 year of age. Furthermore the study showed no difference in need for rescue medication and a lower cumulative morphine dose over 48 hours after surgery. This study shows that IV paracetamol is effective as a primary analgesic or as an adjuvant to morphine infusion after major non cardiac surgery.

Several pain conditions have been shown to have a considerable genetic contribution. More than 350 candidate genes have been identified that may be involved in heritable differences in pain sensitivity and processing. Even though twin studies can help identify if different phenotypes in pain sensitivity are genetically linked, they are unable to identify potential genes involved (17, 18).

Understanding of PK, PD as well as genetic variation concerning pain perception is therefore important to further improve dosing protocols. The perception of pain varies greatly between patients. Besides genetics other factors such as prior opioid use, and opioid tolerance may influence perception of pain.

**Cardiothoracic Surgery**

In cardiac surgery patients postoperative pain is influenced by several factors. These factors are related to the surgical procedure, such as the sternum wound and chest drains. In addition, mechanical ventilation, endotracheal suctioning, and coughing after extubation all may add to the intensity of the postoperative pain. Dislocation of the sternum or the drains can result in serious complications that require repeat surgery.

Based on the general anesthesia guidelines, opioids are considered standard of care in children to prevent and treat postoperative pain after cardiac surgery even though clear PK data in this specific pediatric population is lacking(19). In a large prospective study Howard et al. evaluated effectiveness, morphine requirements and safety of iv morphine in over 10.000 pediatric patients including almost 1000 patients after cardiac surgery in a tertiary care hospital. Patients after cardiac surgery had the highest morphine requirements with an average dose of 23 mcg/kg/h (5-50 mcg/kg/h) but PK data was not collected (20) and it is unclear whether morphine dosing was based on validated pain scores. Lynn at al. describe morphine serum levels of 15-20 ng/ml in infants after cardiac surgery with continuous morphine infusions of 10-20 mcg/kg/h even though there were no reports of analgesic efficacy of these doses (21). These morphine serum levels are typically associated with adequate pain relief (22). However, PD endpoints have not been scored using validated instruments and no PK data have been published (23).

Although morphine requirements and dosing algorithms have been published for post-operative pain relief these studies mostly describe either adult (24)or non-cardiac surgery pediatric patients (15). We know PK is significantly different in children as compared to adults. Moreover, there are several reasons to assume that children after cardiac surgery have different PK and/or PD compared to other types of surgery.

The use of cardiopulmonary bypass (CPB) is one of the main reasons to expect PK changes in this patient population as compared to patients after non-cardiac major surgery. CPB has a profound effect on the pharmacokinetics because of 1) hemodynamic changes, 2) hemodilution, 3) hypothermia, 4) systemic inflammatory reactions, 5) sequestration of drugs. These effects change constantly throughout CPB and some continue to exert influence after the patient has been successfully weaned from CPB (25).

1) During CPB there is a change from pulsatile to non-pulsatile flow with rapid hemodynamic changes. This may change organ perfusion and organ function. Most children after cardiac surgery are dependent on inotropic support and at risk of further hemodynamic instability. Hemodynamic changes affect organ perfusion and ultimately organ function. This will affect drug metabolism. Children with a normal cardiovascular system undergoing surgery seem to clear morphine more efficiently than infants undergoing cardiovascular surgery (26). The authors concluded that maturation processes of the metabolizing enzymes are different for post cardiac surgery patients but might more likely be due to changes in organ perfusion and subsequent changes in metabolizing enzymes. Further evidence suggests that clearance of morphine is significantly lower in children who need inotropic support after cardiac surgery (22). An accurate prediction model for morphine clearance therefore needs to take both type of surgery (cardiac vs non-cardiac) and age into account.

2) On initiation of CPB, prime fluid causes dilution of the patients' blood. Particularly, in small children the addition of the prime fluid can double the patient circulating volume. This causes a shift in the bound and unbound fraction of the drug and a redistribution from peripheral to central compartments. The influence of these shifts depends on the drug properties concerning protein binding, lipophilicity and volume of distribution. Moreover, the patients weight determines the amount of albumin added to the prime fluid, thereby influencing the protein binding of drugs. The added volume during cardiopulmonary bypass will also be influenced by the use of modified ultrafiltration. This modified ultrafiltration will extract fluid from the patients during CPB in order to reduce the circulating volume. Both hemodilution and use of CPB decreases blood pressure and blood flow rate (25). The decreased renal and hepatic perfusion, and hemodilution of red blood cells, may result in decreased elimination of drugs. Finally, hemodilution also causes a shift in acid-base status of the circulating volume affecting bound to unbound drug ratio's which in turn may affect the PK of drugs.

3) Decreased elimination is also strongly influenced by hypothermia, due to temperature dependence of metabolic enzyme function and transporter molecules. The most extreme example of hypothermia is a body temperature of 18° C during deep hypothermic circulatory arrest (25, 27-30). Upon rewarming these metabolic changes are gradually reversed.

4) The systemic inflammatory response that is activated by CPB depends on patient characteristics and of CBP duration. The complex humoral response includes complement, cytokine and coagulation-fibrinolytic activation. The cellular response includes activation of endothelial cells, neutrophils, macrophages, monocytes and platelets. Interesting endogenous molecules in these immune responses are alarmins, that are released upon tissue damage and activate the immune system (31). However, the primary initiation of the alarmins in the immune response are unclear (32). The result of the pro-and anti-inflammatory mediators results in the "systemic inflammatory response syndrome" (SIRS). SIRS can vary from mild to severe, with multiple organ dysfunction. Mild SIRS will have almost no consequences for the treatment of the patient on the ICU. Severe SIRS, however, will have serious consequences for the immediate treatment of the patient and will result in a prolonged stay in the pediatric ICU (25, 33, 34).

The early inflammation phase is triggered by the moment the blood comes in contact with the synthetic circuit of the CPB. The late phase of the inflammation cascade is triggered by ischemia-reperfusion and endotoxemia, following the release of the aortic cross clamping.

Nonspecific factors activating the inflammatory response are hypothermia and transfusion. Auto-transfusion is used in order to reduce the systemic inflammatory biomarkers (35).

In order to reduce the systemic inflammatory response, specific patients may receive a bolus of glucocorticoids at the start of anesthesia. Type, dosing and timing of glucocorticoids vary between hospitals and pediatric data are scarce. The effects of glucocorticoids on the anti-inflammatory response and clinical outcome remain conflicting (36-38). However a recent study in children during cardiac surgery found a temporary, controlled activation of the innate immune system with both strong pro- and anti-inflammatory signals (39).

5) Sequestration of, mainly lipophilic, drugs in het CPB system depends on the different components of the CPB circuit. Studies on midazolam and propofol found that, although sequestrations leads to a decrease of the total serum concentration, the unbound fraction often remains reasonably stable (40). The type of tubing and oxygenator have the most influence on drug sequestration, as demonstrated with propofol and fentanyl (41, 42). The available literature on drug sequestration in CPB is all based on adult CPB circuits. In vitro and in vivo pediatric CPB circuit are currently tested at the Erasmus MC.

Since the publication by the group of Lynn (21, 26) most pediatric cardiac intensive care units use morphine as standard analgesic drug in cardiac surgical patients, sometimes complemented by NSAIDs (in children over 3 months) and often by paracetamol. Because of a lack of evidence on specific drugs, considerable differences in type and dosing of analgesics are observed worldwide. Dosing of morphine, for instance, was found to range from 10 to 80 mcg/kg/h (43).

Regional anesthetic techniques are not used in children during cardiac surgery. Studies in this field are of limited size and the complications of regional techniques are rare, but severe (44).

Finally, the type of surgical procedure may influence the PK of drugs. More complex congenital cardiac defects may not only have an influence on systemic circulation influencing PK of drugs itself, but will also lead to longer CPB run times with higher risk for severe SIRS and hemodynamic instability. This said there is currently no literature suggesting a difference in sedative or analgesic requirements based on type of congenital defect or surgical procedure. Furthermore current hospital guidelines on analgesia and sedation in children after cardiac surgery do not distinguish between type of surgical procedure. An extensive literature search did not yield a relation between operative procedure and dosing and type of analgosedation.

**CPB and pharmacodynamics**

There are reasons to expect changes in PD, i.e. the sensitivity in terms of efficacy or safety to a certain drug concentration, in patients undergoing CPB for cardiac surgery. There is increasing evidence that up to 50% of neonates with a CHD have pre-operative cerebral lesions (45-47). Experimental data shows that CPB causes cerebral swelling potentially influencing the PD or efficacy of drugs (34). Also, cerebral injury has been shown, due to microemboli, in adults. The amount of microemboli are dependent on the duration of CPB (48). Cerebral injury may also occur in children who needed deep hypothermic circulatory arrest to correct their congenital malformation. White matter injury was the most common injury in children after deep hypothermia, independent of different perfusion technique (low flow, or circulatory arrest) (45). It is unclear if and how this may affect the pharmacodynamic relation of drugs.

**Cardiac surgery, CPB and influence on stress hormone levels**

CPB elicit a critical illness stress response in infants, which is associated with changes in the hypothalamic-pituitary-adrenal (HPA) axis. There is limited understanding of the axis function during and after cardiac surgery in children (49). However, in the acute phase of critical illness, circulating cortisol increases in response to acute illness or trauma, assumed to be driven by a several-fold increased adrenocortical cortisol production evoked by increased CRH and ACTH release. Because of a decrease in

albumin and corticosteroid-binding globulin levels, a proportionally much higher increase of circulating free cortisol is present (50). When critical illness persists, a steep fall in plasma ACTH occurs after a short lasting elevation, whereas plasma cortisol concentrations remain high, due to a pronounced suppression of cortisol breakdown by a reduced expression and activity of the cortisol metabolizing enzymes (51). In animal studies and healthy subjects, effects of opioids on the HPA axis appear to be mediated via both the hypothalamus and pituitary, but also via opioid receptors on the adrenal gland (52). However, in critically ill adults, Peeters et al. described a dose-dependent suppressive effect of opioids on plasma cortisol – but not on ACTH (53). This suggests a drug effect not mediated centrally via ACTH, but rather peripherally by a direct or indirect action on the adrenal cortex.

**Morphine**

The elimination of morphine is mainly through glucuronidation by urine diphosphate glucuronosyltransferase (UGT) 2B7. Morphine clearance therefore directly reflects the formation of its two major metabolites morphine-3-glucuronide (M3G) and morphine-6-glucuronide (M6G). Both metabolites are cleared through renal elimination, and a reduced renal function can lead to accumulation of the metabolites. Evidence suggests that clearance of morphine is significantly slower in children who need inotropic support after cardiac surgery (22). Since the use of post-operative inotropic drugs implies a compromised cardiovascular status, this could have an impact on morphine metabolism (22). M3G and M6g are pharmacologically active, thus accumulation is of clinical importance. Accurate predictions of the metabolites clearance in children of different age groups after cardiac surgery have not yet been established. The existing prediction models after cardiac surgery, from both the traditional and population-based approach focus on the total morphine clearance (11).

Morphine is known to have unwanted adverse drug reactions. This is a particular problem in these hemodynamically unstable children. Hypotension and respiratory depression can lead to delayed recovery and prolonged PICU stay (54, 55). Other adverse effects of morphine are intestinal obstruction, which mainly occurs in younger children and nausea, vomiting and itching mainly occurring in older children (7). Alternative analgesic drugs such as paracetamol or nonsteroidal anti-inflammatory drugs (NSAIDs) may reduce opioid requirement, improve analgesia and reduce the adverse effects (56). These concerns lead to the earlier mentioned randomized controlled trial of IV paracetamol vs continuous IV morphine.

**Paracetamol**

Paracetamol is the most frequently used antipyretic and analgesic agent in children for the treatment of fever or acute pain. The working mechanism of paracetamol is not yet fully understood. Considering the analgesic, antipyretic and weak anti-inflammatory actions it is likely due to the inhibition of prostaglandin synthesis in the central nervous system and in peripheral tissue (57). The analgesic effect is likely due to interaction with the serotonergic system. Co-administration of receptor 5-HT3 antagonists completely blocks the effect of paracetamol (58). Several studies have determined paracetamol metabolism in adults and children (59-61).

Glucuronidation is the major pathway of paracetamol metabolism (50-60%), with an additional contribution of sulfation (25-44%) and oxidation (2-10%). Glucuronidation and sulfation result in inactive and non-toxic end products, which are excreted by the kidney. In the oxidation pathway the hepatic cytochrome P450 forms mainly catechol and NAPQI (N-acetyl-p-benzo-quinone imine). NAPQI is conjugated by glutathione into cysteine and mercapturic acid metabolites and eliminated in the urine or bile (61). Interference with the conjugation of NAPQI may have clinical consequences. NAPQI is toxic and in case of overdose causes mitochondrial dysfunction and centrilobular necrosis in the liver (60). In children the glucuronide formation clearance, but not the sulphate formation increases with age. A mature value for clearance was found in children who were approximately 8 months old (62). Several studies show that, when used in therapeutic doses in children without liver dysfunction the safety profile is excellent (61). In the past, concern has been raised about the association between paracetamol use in early life and the development of asthma later in childhood (63). Recent evidence shows that this association is entirely due to confounding by indication (64).

Enteral or rectal dosing is sufficient for mild to moderate pain. However, after major surgery rectal and oral administration of paracetamol proved to be insufficient to reach a therapeutic level in 22% of all patients. Furthermore there was a wide range in drug levels (65). Moreover, rectal paracetamol does not reduce the morphine consumption after major surgery in young children (66). Potentially, intravenous paracetamol performs better in case of severe or acute pain. The penetration of IV paracetamol through an intact blood brain barrier has been shown in children who required a spinal catheter for surgical procedures on the lower body. Paracetamol was detected in the spinal fluid within 5 minutes after injection of an intravenous bolus, with the highest concentration at 57 minutes after injection (57). The rapid penetration of paracetamol in the central nervous system contributes to the fast onset of the analgesic and antipyretic effect. In recent studies analgesic effect was seen within 15 minutes of administration in adults (67). Fever reduction was seen within 30 minutes (68).

Worldwide IV paracetamol is given in 3-4 dosages daily and not as continuous infusion. Even in adults no data are available on the safety and efficacy profile of continuous paracetamol infusions, making this approach ethically not justifiable for studies in children at this stage .

Data on paracetamol pharmacokinetics in children have been studied in several groups, including our own. Pharmacokinetics of IV paracetamol in children until the age of 16 years have been described with non-linear mixed-effect modeling, using weight, clearance and volume parameters in a two compartment model, even though no children undergoing cardiac surgery were included in this study (69, 70).

**Pharmacogenetics**

The interindividual variability observed in the analgesic requirement is the consequence of the diversity in evoked response to the painful stimuli in an individual and the sensitivity to the drug. The amount of pain experienced and the response to analgesia is depending on physiological, psychological and environmental factors as well as genetic predisposition. Up to date a large number of candidate gene studies has illustrated associations between genetic variants with opioid response (71, 72) and

paracetamol efficacy (73). The genetic impact can arise from polymorphisms in genes that alter drug levels (PK) such as metabolizing enzymes and transporters or in variants from genes relevant for the nociceptive function such as the mu-opioid receptors.

As discussed previously one of the PK genes relevant for the hepatic elimination of morphine is *UGT2B7*. The -842G>A (rs7438135) polymorphism of this phase-II enzyme has been related with altered morphine, M3G and M6G plasma levels in adults (74), which later was replicated in a pilot cohort with preterm newborns (75). More recently other PK related genetic variants for morphine (*ABCC3* and *OCT1*) were also associated with morphine PK and PD (76-78)For paracetamol the *3 polymorphism in the cytochrome P450 enzyme *CYP3A5* has been associated with an enhanced NAPQI formation (79), whereas a negligible amount of focus has been on CYP2E1, the more relevant enzyme in the formation of this toxic metabolite (80). Also a few studies have been published focusing on the phase-II metabolism of paracetamol (73). A recently published meta-analysis illustrated that the highly investigated polymorphism 118A>G (rs1799971) in the mu-opioid receptor (*OPRM1)* causes variability in opioid requirement after surgery.

**Current guidelines**

There is only limited information on the PK And PD of morphine in children after cardiac surgery. Studies show increased dosing of morphine but lack clear PD endpoints or detailed PK data (20, 21). PK data from a cohort of 40 pediatric patients after cardiac surgery showed an increased volume of distribution with a reduced overall clearance (81, 82). This is in line with adult data (23, 83).

Due to this lack of good quality evidence there is no clear consensus regarding analgosedation after cardiac surgery in children and Guidelines are lacking. Our group has conducted a survey to evaluate analgosedation practices in several large pediatric cardiac surgery centers. In this survey, all four pediatric cardiac surgery centers in the Netherlands (Rotterdam, Leiden, Utrecht and Groningen) and 11 international pediatric cardiac surgery centers participated. In total, the participating centers treat over 3000 pediatric cardiac surgery patients annually. The survey revealed that 8 different opioids were used in the 15 participating centers. Also, dosing regimens for the different drugs were found to vary per center; for instance morphine was administered as continuous infusion from 5 to 100 mcg/kg/h in infants and young children younger than 4 years of age. None of the centers distinguish between type of cardiac surgical procedure when prescribing analgesic therapy.

Next to opioids, most centers use additional analgesia, such as paracetamol, diclofenac and ibuprofen in varying doses without appropriate studies showing whether this multimodal approach is of any benefit. The results of the survey are summarized in Appendix 1.

**In conclusion**

There are no clear guidelines regarding analgesia after cardiac surgery in children. The current standard of care is morphine, with dosing ranging from 10-80 mcg/kg/h worldwide. However, morphine has unwanted side effects, making an alternative analgesic agent desirable. Intravenous paracetamol is a potential replacement as the primary analgesic in children after cardiac surgery and has an excellent

safety profile. Moreover, there is substantial evidence from major non-cardiac surgery that IV paracetamol is effective in reducing morphine doses in children. Therefore, this study will focus on the reduction, or replacement, of morphine by intravenous paracetamol in children aged 0-36 months after cardiac surgery. The results of this study will form the bases of a new pain management algorithm and will be implemented at the participating ICUs, resulting in an evidence-based nationwide guideline on post-operative pain after cardiac surgery in infants aged 0-36 months.

**Hypothesis**

It is our hypothesis that intermittent IV paracetamol is effective as the primary analgesic drug in post cardiac surgery patients up to 3 years of age and that the use of IV paracetamol will reduce overall morphine requirements.

**Line of research**

This research is strongly embedded in current Erasmus MC Sophia research collaborations ensuring excellent support for this study. The pediatric intensive care department has a long standing experience in conducting trials, including pediatric pharmacokinetic pain studies. A previous trial conducted at this department evaluated the optimal way of administration of morphine in children between 0-3 years of age for postoperative pain relief (MEC 1994-166). We have also conducted a previous study on morphine versus paracetamol after non-cardiac major surgery in children (MEC 2007-355), of which the results are recently published in JAMA (16). Intravenous morphine versus intravenous paracetamol in children on extracorporeal membrane oxygenation (ECMO) was also studied in this department (MEC 2009-334).

In recent years several PhD students have obtained their doctoral degree on research in the pediatric cardiac surgery population in the Wilhelmina Children’s Hospital at UMCUtrecht. The cerebral perfusion during neonatal cardiac surgery has been investigated (MEC 08-090) (45, 84). Also, the pro-and anti-inflammatory reaction after cardiac surgery in children has been studied. And a model for inflammatory regulation at the PICU has been established (MEC 04/144) (85-87). A new line of research in pediatric cardiac surgery patients is primarily aimed at better understanding the role of alarmins as mediator in systemic inflammatory reactions taking place during and after surgery. An understanding of how the immune response is activated by (surgically induced) trauma may provide the insights necessary to improve the care for patients after the surgery (32, 88). Moreover, alarmins may serve as a potential target for biological therapy (89).

The Dutch Expertise Center for Pharmacotherapy in Children operates from the Erasmus MC Sophia. We also have a strong and long collaboration with the Leiden Academic Centre for Drug Research in Leiden. Our group produced over 140 peer reviewed manuscripts and 14 PhD students graduated on the topic of analgesics and sedation.

Research results from previous studies have been implemented at the pediatric intensive care unit through guidelines, which are in use in daily practice. This has also been achieved for other projects who

received funding from ZonMw, such as the study by Ceelie et al (16). The guidelines will be implemented at all the pediatric ICU departments that participate in the study.

## 2. Objectives

**Primary objective:**

To test the hypothesis that analgesia with intermittent intravenous paracetamol will lead to a morphine sparing effect of at least 30% as compared to model-based continuous iv morphine infusion during the first 48 hours after cardiac surgery in infants aged 0-36 months.

The primary outcome measure is the weight-adjusted cumulative morphine consumption (mcg/kg) in the first 48 hours post-operatively.

**Secondary objectives:**

1. Incidence of adverse drug reactions
    a. hemodynamically: hypotension or bradycardia, with the need for intervention by means of medication or a fluid bolus.
    b. Decreased gastro-intestinal motility or intestinal obstruction not directly related to the underlying diagnosis and not previously existing, with the need for intervention.
    c. Vomiting.
    d. Number of re-intubations.
    e. Pediatric delirium as measured by the SOS-PD-scale.
2. Non-inferiority analysis of comparing patients with one or more NRS pain scores ≥4 between groups.
3. DNA analysis will be performed to evaluate the effect of gene polymorphisms on the PK of analgesic medication.
4. Concomitant use of sedatives.
5. The number of hours on ventilation.
6. The length of PICU stay.
7. Role of alarmins in the systemic inflammatory response (only at Wilhelmina Children's Hospital, UMCUtrecht).
8. To develop a population PKPD-based post-operative pain management algorithm based on the results of this trial.

## 3. Study design

This study is a multi-center, prospective, randomized, double blind study comparing the total amount of morphine needed in children aged 0-36 months who receive either intermittent intravenous paracetamol intravenous + morphine rescue or a continuous iv morphine infusion + morphine rescue, for the first 48 hours after cardiac surgery.

**Blinding**

The randomization schedule is kept by the local hospital pharmacist to ensure blinding until the end of the study. Each hospital pharmacy will participate in the study.

In case of a medical emergency, for instance rise in liver enzymes, or in case the installed treatment is not adequate in terms of sufficient pain relief the hospital pharmacist on call can be consulted what medication was administered to a patient to take appropriate action.

In case of post-operative fever or re-operation the study medication will be stopped. This is described in the section 6.4 ‘withdrawal of individual subjects’. The pharmacist does not have to reverse blinding of the study medication to facilitate adequate treatment.

## 4. Study population

### 4.1 Patients

Patients eligible to participate in the study will be infants and children aged 0-36 months admitted to the ICU after cardiac surgery with the use of the CPB. Patients will be stratified per participating ICU.

### 4.2 Inclusion criteria

- Informed consent,
- Neonate / infant aged 0-36 months,
- Cardiac surgery with the use of CPB.

### 4.3 Exclusion criteria

- No informed consent
- Known allergy to or intolerance for paracetamol or morphine,
- Administration of opioids in the 24 hours prior to surgery.
- Hepatic dysfunction defined as three times the reference value of ALAT/ASAT.
- Renal insufficiency defined as Pediatric RIFLE category - injury, defined as estimated creatinine clearance reduced by 50% and urine output <0.5 ml/kg/h for 16 hours.

**4.4** Pre-operative liver and renal function will only be screened as part of standard care or when there is a clinically relevant reason to evaluate liver and renal function. No additional screening will be performed only for the study purposes. No additional blood draws will take place for this evaluation. **Sample size calculation**

The primary outcome measure is the weight-adjusted cumulative morphine consumption (mcg/kg) in the first 48 hours post-operatively. We estimate that the required morphine dose in the paracetamol group can be reduced by at least 30% compared to the morphine group in the first 48 hours after surgery.  This is in line with the outcome of a previous study we conducted in a similar patient group with similar study medication (MEC 2007-355) (16).

The power analysis is based on a comparison of the primary outcome between groups using a Mann-Whitney test (i.e. the unstratified version of the Van Elteren test). A simulation study was done for this power analysis using data on the cumulative morphine dose from a previous study with comparable morphine dosing (16). Based on this data set, the median cumulative morphine dose was 357 mcg/kg (IQR: 220-605) in the control group, and we assumed that this morphine dose will be reduced by 30% in the intervention group. The simulation study showed that using a two-sided significance level of 5%, 86 patients per group will be required to obtain a power of 95%. To account for the effects of stratification by centre in the Van Elteren test and missing data, we will include 104 patients per study arm, 208 in total. We expect this sample size to also be sufficient to assess secondary outcomes.

These observations come from a population aged 0-12 months, but we do not suspect larger differences for our older infants (and even smaller differences).

As described above, the primary endpoint of the study is the total amount of morphine administered in the first 48 hours after surgery. Any additional morphine given will be based on NRS scores of 4 and higher. In a secondary, noninferiority analysis, we will therefore also compare the number of patients with one or more NRS scores of 4 and higher between the two groups, using a non-inferiority margin of 20%. Non-inferiority will be assessed using a one-sided 97.5% confidence interval for the difference in the percentage of patients with at least one NRS score of 4 or higher between the paracetamol group and the morphine group, and non-inferiority will be proven if the upper limit of this confidence interval is lower than 20%. The confidence interval will be calculated using the method of Klingenberg (Stat Med 2014), with adjustment for center. Using data from our previous study (16) on Paracetamol and morphine it is estimated that 60% of all patients will have one or more NRS scores >3. Using a simulation study, we calculated that to detect non-inferiority with a power of 75%, 200 patients (100 per group) are required. Even though the power is 75%, this is considered to be sufficient for this secondary endpoint. This secondary non-inferiority analysis can be done with the 208 patients per group that resulted from the samples size calculation for the primary endpoint.

## 5. Treatment of subjects

### 5.1 Investigational product/treatment

All patients will receive a loading dose of morphine 100 mcg/kg in the OR which is based on the results of the survey (Appendix 1). After the loading dose, patients are randomized to either receive a morphine continuous infusion or intermittent intravenous paracetamol. Intravenous paracetamol and intravenous morphine are both registered for the Dutch market for pain relief. There is extensive clinical experience with the use of paracetamol and morphine in neonates and children. Patients in the intervention group receive intermittent intravenous paracetamol after the loading dose of morphine 100 mcg/kg.

**Control group**

Patients in the control group will receive a continuous morphine infusion. Based on our PK data on morphine in non-cardiac pediatric patients (10, 14), adult post cardiac surgery patients (23) as well as the data on 40 pediatric post cardiac surgery patients we developed a new morphine dosing regimen for neonates and children aged 0 to 36 months. The morphine dosing regimen is added to the research protocol as appendix 2.

**Use of co-intervention**

Standard postoperative care is given to all patients with analgesics recue medication described under 5.2. The use of sedatives is standardized for the participating ICUs according to the treatment algorithm used at the Erasmus MC-Sophia (Appendix 3).

### 5.2 Rescue analgesic medication

Rescue morphine is administered whenever the Numeric Rating Scale (NRS) pain is equal to or greater than 4, as is part of standard clinical care (see our postoperative pain protocols, Appendix 4).

Additional morphine will be administered every 10 minutes whenever needed, with a maximum of three times per hour. Standard additional dose of morphine is 10 mcg/kg for individuals aged <10 days postnatal age and 15 µg/kg for individuals aged ≥10 days .

Ten minutes after each extra dose of morphine, pain is re-assessed. If there is no improvement in the scores (i.e. NRS ≥ 4) after three additional (rescue) doses, a continuous morphine infusion is started in a separate pump (to ensure blinding). Whenever pain is not responding to the extra morphine boluses and the additional continuous morphine infusion in a maximum dose of 30 mcg/kg/h, fentanyl is started at 1-2 mcg/kg loading dose and 1-3 mcg/kg/h continues infusion. At the start of fentanyl, morphine will be discontinued. In case of discomfort (Comfort-B score >22or COMFORT-B score between 11 and 22 but NISS suggesting undersedation) midazolam is started (Appendix 3). Sedation protocols are already very comparable between the participating ICUs, regarding the primary sedatives. Standardized sedation will be part of the treatment protocol (see sedation protocol, appendix 3). This treatment algorithm is

similar with the one used in the recent study comparing morphine and paracetamol in non-cardiac patients (MEC 2007-355) (16).

In both groups, continuous morphine infusion (if started) will be decreased in the second 24 hours depending on the NRS and COMFORT-B score.  (see appendix 4)

## 6. Methods

### 6.1 Main study parameter/endpoint

Cumulative morphine dose over 48 hours in mcg/kg.

#### 6.1.1 Secondary study parameters/endpoints (if applicable)

- Level of pain assessed by validated PD instruments until 48 hours after stop study medication
- Variation in nurse and parent evaluation of pain or discomfort
- Incidence of adverse drug reactions
- Incidence of concomitant use of sedative
- Hours on ventilation
- Incidence of over- and undersedation
- Incidence of withdrawal syndrome and pediatric delirium
- The length of PICU stay
- Observe the changes in the HPA-axis as a response to critical illness .
- Assess the influence of iv morphine on the changes in the HPA axis in critical ill infants after cardiac surgery.

Other endpoints

- Use of corticosteroids
- Parents postoperative pain measurement two days after discharge (PPPM-SF)
- Role of alarmins in the systemic inflammatory response (only at Wilhelmina Children's Hospital, UMCUtrecht).

#### 6.1.2 PD outcome measurements

PD outcomes will be measured using validated instruments. Both analgesia and sedation need to be assessed (Appendix 5). Pain and discomfort can interact, making accurate assessment difficult. Therefore, the use of concomitant sedative drugs will be standardized in the participating ICUs. The COMFORT-Behavior scale (COMFORT-B scale) is a pain and distress assessment instrument that asks observers to consider the intensity of six behavioral manifestations: Alertness, Calmness, Respiratory response (for mechanically ventilated children) or Crying(for spontaneously breathing children), Body movements, Facial tension and Muscle tone. For each of these items, five descriptions, rated from 1-5, are provided reflecting increasing intensity of behavior in question. Summating the ratings of the six behavioral manifestations leads to a score ranging from 6-30. The COMFORT-B scale has been extensively validated in postoperative infants with and without Down syndrome and in infants after major cardiac surgery. The COMFORT-B scale has also been validated in the use in pre-verbal children

(90-93). These instruments have also been used in several previous studies, including the one published by Ceelie, JAMA 2013 (MEC 2007-355) (16).

The Numeric Rating Scale pain (NRS pain) is used in conjunction with the COMFORT-B scale to represent the rater's expert opinion (94).

Parents will be able to participate in rating the pain in their children, using the Numeric Rating Scale Pain (NRS-pain) (Appendix 6). This scale asks the parents to rate pain with a number from 0 (no pain) to 10 (worst pain possible). This will be used alongside the nurses NRS and COMFORT-B scale. In the current study we will ask the parent to apply the NRS after the nurse has entered her NRS in the patient data management system. When the two score differ significantly (one of the two applies a score> 3 and the other not) they will discuss the reason for their score. Both nurses and parents will receive a very short instruction explaining the different factors that should be take into account when assigning the NRS.

Parents participation will be on a voluntary basis. In agreement with the patient associations we believe that some parent may want to participate more actively in the detection and rating of pain in their children. If parents indicate that active participations is not wanted anymore, they will be able to stop at any time. Variation in nurse and parent evaluation of pain or discomfort will be analyzed as a secondary endpoint.

The Nurse Interpretation of Sedation Score (NISS) has been validated for this age group and represents the caregiving nurse's expert opinion scored as 1= undersedation, 2= adequate sedation, or 3= oversedation (94).

Follow-up will consist of the “parents postoperative pain measurement” (PPPM-SF) to complete after discharge from the hospital (Appendix 7). This will record the pain perception and management at home after surgical intervention (95). Parents will be called two days after discharge to inform after the child health and to take the questionnaire. Also parents will have the change to ask questions considering the daily care of the child. The patient association representatives stated that parents often have questions about the daily routine that they did not ask in the hospital. Calling after discharge provides the researcher the change of the follow-up and allows parents to gain additional information about their daily routine. The centre specific researcher will call the parents and will forward any medical related questions to the designated physician.

Opioid withdrawal and pediatric delirium and can cause problems on the ICU. Withdrawal can be difficult to treat and sometimes it takes months before the patient is completely free of symptoms. However, withdrawal mostly occurs after a prolonged opioid use of at least five days. Delirium is observed frequently after cardiac surgery, occurring shortly after surgery. Delirium has been shown to increase post-operative morbidity (78, 96).

The Sophia Observation withdrawal Symptoms-scale (SOS) has been validated to detect withdrawal syndrome in critically ill children. It contains 15 items that are scored either not present (0) or present (1). A score of 4 or higher suggests withdrawal syndrome (97).

Pediatric delirium in our study will be assessed with the SOS-PD (Appendix 8). The SOS-PD consists of 17 items representing (behavior) symptoms pediatric delirium with explanations where necessary. The maximum total sum score is 17 points. A total score of at least 4 was used as a cut-off for delirium or when the item “hallucination” is scored positive. According to standard local protocol a pediatric psychiatrist will be consulted in case of delirium. Treatment of delirium will be done according to local protocol.

Severity of illness will be estimated with the validated Pediatric risk of Mortality-lll score (98) together with the more specific Risk Adjusted Classification for Congenital Heart Surgery (RACHS-1) score (99). All Dutch hospitals use the Aristotle score to classify the congenital cardiac surgery patients. This Aristotle score will also be used to estimate the severity of surgical procedure and to compare the two groups with respect to the surgical intervention (100, 101). The PELOD-2 score will be used to assess the severity of cases of Multiple Organ Dysfunction Syndrome in the PICU on day 0, 1 and day 2 (102).

An advisory board is already established composed of representatives from two patient associations (*Stichting Kind en Ziekenhuis, en Patientvereniging Aangeboren Hartafwijkingen*). Also prof. John van den Anker, a professor in neonatology and clinical pharmacology and dr. Maarten Witsenburg a pediatric cardiologist partake in the advisory board. The advisory board has been involved in the design of the study.

Adverse drug reactions:

Respiratory:

1. Number of re-intubated patients during the study period in each group.
2. Duration of mechanical ventilation in hours in each group.
3. Number of apnea episodes as defined by SpO2 <94% or respiratory rate <20/min longer that 30 seconds.

Hemodynamically:

1. Hypotension with the need for vaso-active medication or fluid boluses.
2. Bradycardia (>30 seconds).

Gastro-intestinal:

Decreased gastro-intestinal motility or intestinal obstruction not directly related to the underlying diagnosis and not previously existing, with the need for intervention. Pre-operative the intestinal status will be noted. Post-operative, the patient will be examined daily for signs of obstipation, nausea and vomiting.

Other:

Regular laboratory screening is part of the standard care on the PICU. Specific laboratory values will be registered in the study database. This will give informative on respiratory and hemodynamic status, liver

and renal function and inflammation. Post-operative liver and renal function will only be screened as part of standard care or when there is a clinically relevant reason to evaluate liver and renal function. No additional screening will be performed only for the study purposes. No additional blood draws will take place for this evaluation.  Also vital parameters are registered during PICU stay in electronic patient chart (PDMS), which all participating centers use.

### 6.1.3 PK analysis and Blood sampling regimen:

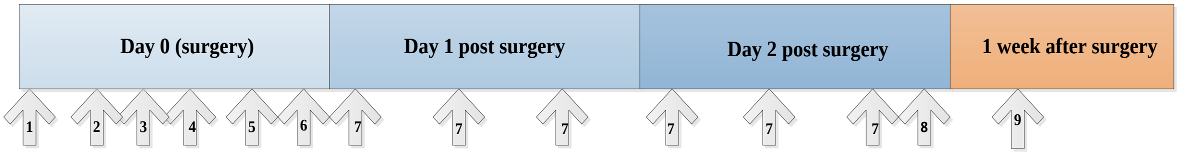


1. After insertion of arterial line, sampling for DNA analysis (1 ml). *Only at Erasmus MC-Sophia Children's Hospital and UZ Leuven: sampling for ACTH and cortisol (1 ml). Only at Wilhelmina Children's hospital: sample for alarmins analysis (0.5ml)*
2. Stop cardiopulmonary bypass, first PK blood sample (1 ml).
3. Loading dose morphine 100 mcg/kg (1 ml). *Only at Erasmus MC-Sophia Children's Hospital and UZ Leuven: sampling for ACTH and cortisol (1 ml).*
4. Start study medication (IV paracetamol or IV morphine); 2 hourly pain and sedation assessment commences (1 ml).
5. Second PK blood sample (T= 30-60) (1 ml).
6. Third PK blood sample (T= 180-240) (1 ml). *Only at Wilhelmina Children's hospital: sample for alarmins analysis (0.5ml).*
7. Repeated blood measurements during morning, afternoon and evening standard rounds (1 ml). *Only at Erasmus MC-Sophia Children's Hospital and UZ Leuven, first and second morning after surgery: sampling for ACTH and cortisol (1 ml). Only at Wilhelmina Children's hospital: sample for alarmins analysis 24 hours after surgery (0.5ml).*
8. End of study 48 hours after start study medication(1 ml). *Only at Wilhelmina Children's hospital: sample for alarmins analysis (0.5ml)*. Follow up of pain and sedation assessment during hospital stay (PICU and general ward).
9. Two days after discharge short telephone interview

Blood samples will be drawn for PKPD analysis, using an indwelling arterial catheter. Blood samples will be taken at three standard moments during the day, after admission to the ICU. Also, samples will be taken before and after changes in the dose of analgesic medication. The PK sampling regimen uses a advised timeframe per sample to ensure a fairly regular distribution of samples. Also, this timeframe enables the PDMS system to send an order to the nurse to draw blood for the PK samples. However, The PK analysis program NONMEM does not use timeframes, it uses the exact time of the blood draw and distribution thereof does not have to be regular. Therefore, if the PK samples are taken outside the timeframe they are equally useful for PK analysis.  Considering this, no violation-to-protocol or protocol deviation report has to be made if a PK sample is taken outside the timeframe.

Because of ethical restriction not more than 5% of the total blood volume will be drawn from the patient. Prior to the start of the study this will be determined. PK samples will thus be obtained by sparse sampling, with a minimal burden to the individual patients. Using population pharmacokinetics analysis the data points from the individual subjects will be combined to form solid pharmacokinetic

data on morphine and paracetamol and their metabolites, since these metabolites are biologically active. The population PK analysis will be done using non-linear mixed effect modelling (NONMEM).

Cortisol and ACTH blood sampling for analysis of the HPA-axis will only take place at Erasmus MC-Sophia Children's Hospital and UZ Leuven. Blood samples will be taken during the standard morning round, using the same indwelling arterial catheter. These additional blood samples will not exceed 2-4ml in total. Including these 2-4 ml, the total blood sampling will be within the range of less than 5% of total blood volume. Blood samples will be collected in prechilled ethylenediaminetetraacetic acid (EDTA) 1ml tubes, immediately placed on iced, centrifuged at 4°C, and then stored at -80°C until assay.

Blood sampling for analysis of the alarmins will only take place at the Wilhelmina Children's Hospital. Blood samples will be simultaneously with PKPD blood samples, using the same indwelling arterial catheter. These additional blood samples will not exceed 2 ml in total. Including these 2 ml, the total blood sampling (PKPD and alarmins) at the Wilhelmina Children's Hospital will be within the range of less than 5% of total blood volume.

DNA analysis will be performed to evaluate interindividual variability drug responses . Genetic variability of morphine and paracetamol will be the main focus.

Urine samples will be collected in portions every 6 hours from the indwelling catheter. Morphine metabolites will be determined.

### 6.2 Randomization, blinding and treatment allocation

Blocked randomization with randomly chosen block sized and stratification by center will be used. The randomization schedule will be kept in the local pharmacy at every center. The pharmacist is the only person to have access to the randomization schedule to ensure concealed allocation. The randomization schedules are made by the study's biostatistician. Study medication will be prepared at the participating centers. By the start of the trial all centers will have the mandatory certificates concerning the preparation and labelling of the study drugs. We will use standard morphine and paracetamol formulations.

The randomization schedule is kept by the pharmacist to ensure blinding until the end of the study. In case of a medical emergency the pharmacists can be consulted what medication was administered to a patient.

Blinding is assured by the use of NaCl 0.9% continuous infusion (in the paracetamol group) or NaCl 0.9% bolus (in the morphine group).

If discharge from the ICU occurs within 48 hours after surgery, the study medication will be continued on the ward. The arterial catheter will be removed at discharge from the ICU. PK sampling on the ward will only be done simultaneously with routine blood examination. The patient will not suffer additional punctures. At 48 hours after surgery, the study medication will be changed to open label paracetamol and rescue morphine if needed.

## 6.3 Study procedures

**Anesthesia**

Anesthetic management during surgery is known to the investigators and will be standardized as much as possible. A blood sample from the patient will be taken at time T=0. Unexpected differences found at later time points can be linked to differences in anesthetic management. Anesthetic management is described in more detail in the appendix (appendix 9).

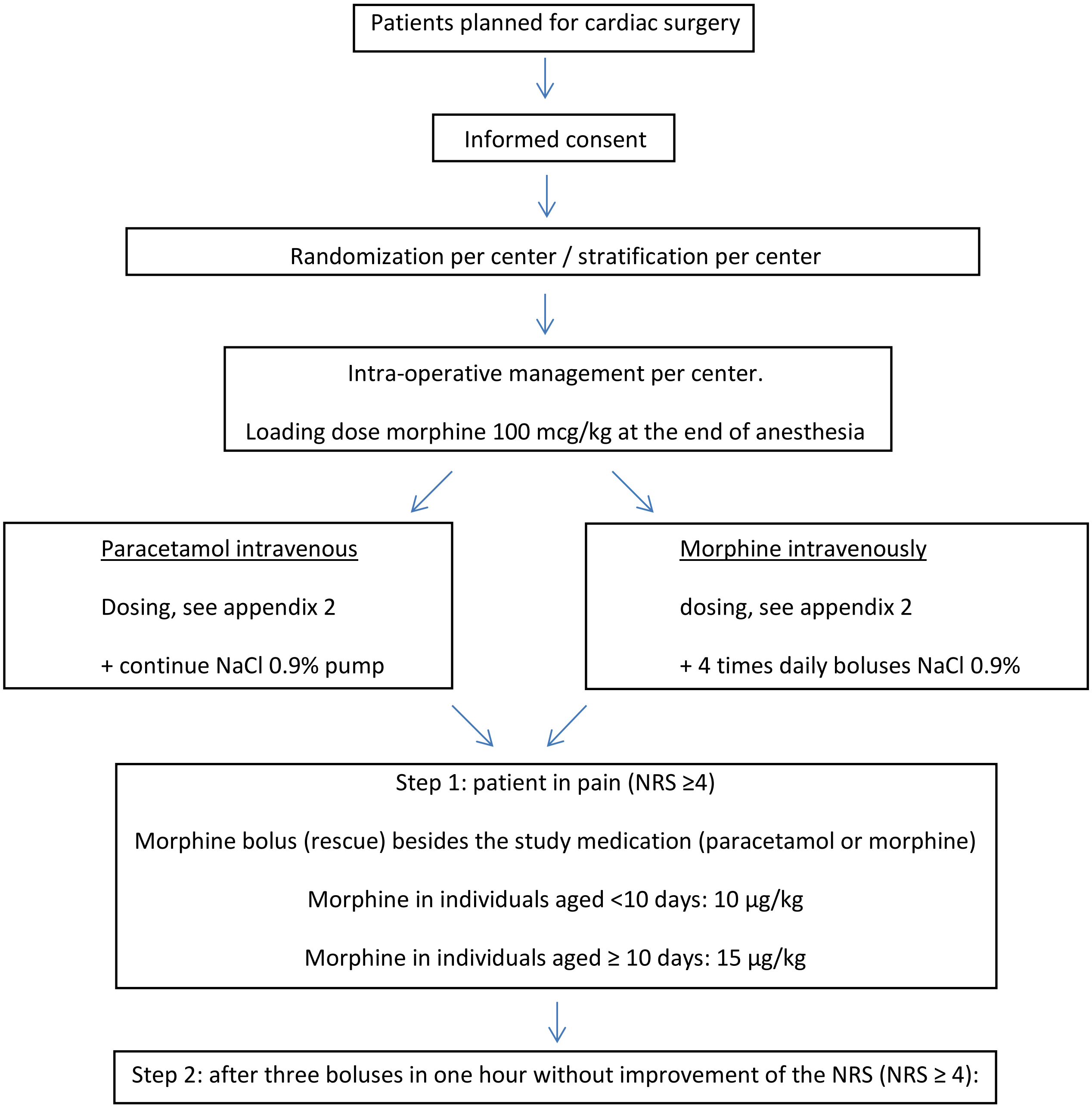

Give morphine 100 μg/kg loading dose and continuous infusion starting at 10mcg/kg/h, infusion can be increased until 30 mcg/kg/h (treatment protocol appendix 4)

(using an extra pump to ensure blinding)

In addition to study medication (paracetamol or morphine)

Repeat step 1 and 2 (if NRS ≥ 4)

↓

Consequently: maximum morphine background reached after increasing the morphine two times besides the study medication

↓

Insufficient pain relief with maximum morphine background infusion:

start fentanyl (VAS ≥ 4). fentanyl is started at 1-2 mcg/kg loading dose and 1-3 mcg/kg/h continues infusion

or midazolam (in case of discomfort) (Comfort-B score ≥ 17). Midazolam loading dose 0.1 mg/kg, infusion start at 100 mcg/kg/h, increase to 300 mcg/kg/h

↓

In case of insufficient pain relief with maximum additional medication:

collecting patient characteristics

↓

END of study;

48 hours postoperative. Change from study medication to open label paracetamol with rescue morphine, if needed.

### 6.4 Withdrawal of individual subjects

Patients can be withdrawn from the study at any time by the investigator or the treating physician. The intention-to-treat analysis will include all subjects. The subjects that have been withdrawn during the study will be included only for the time period in which they have participated. The cumulative morphine requirement will be calculated for the time that the patient participated in the study.

#### 6.4.1 Specific criteria for withdrawal

- Withdrawal of informed consent.
- The patient shows signs of hypersensitivity or an allergic reaction to either morphine or paracetamol.
- If there is a need for a re-operation within 48 hours after the initial surgical procedure.
- In case of ECMO treatment after surgery.
- If there is hepatic dysfunction defined as three times the reference value of ALAT/ASAT.
- Renal insufficiency defined as RIFLE category injury
- Use of muscle relaxants after surgery for the duration of 3 hours of longer.
- Temperature of 38.5 after surgery for the duration of 6 hours or longer.

### 6.5 Replacement of individual subjects after withdrawal

Individual subjects will not be replaced after withdrawal.

### 6.6 Follow-up of subjects withdrawn from treatment

All patients will be followed during their stay in the PICU with a minimum of 48 hours. After discharge of the PICU the follow-up will be continued at the Medium Care department. We expect that some patients will be discharged from the PICU within 48 hours, depending on the cardiosurgical procedure.

### 6.7 Premature termination of the study

The Data Safety Board will monitor the need for rescue morphine. If the need for rescue morphine is too high for the paracetamol group, the study will be stopped.
The Data Safety Monitoring board has already been installed. The trial design has been submitted and evaluated by the DSMB in the first meeting.

## 7. Safety reporting

### 7.1 Section 10 WMO event

In accordance to section 10, subsection 1, of the WMO, the investigator will inform the subjects and the reviewing accredited METC if anything occurs, on the basis of which it appears that the disadvantages of participation may be significantly greater than was foreseen in the research proposal. The study will be suspended pending further review by the accredited METC, except insofar as suspension would jeopardise the subjects' health. The investigator will take care that all subjects are kept informed.

### 7.2 AEs and SAEs

#### 7.2.1 Adverse events (AEs)

Adverse events are defined as any undesirable experience occurring to a subject during the study, whether or not considered related to (the investigational product / the experimental intervention). All adverse events reported spontaneously by the subject or observed by the investigator or his staff will be recorded.

#### 7.2.2 Serious adverse events (SAEs)

A serious adverse event is any untoward medical occurrence or effect that at any dose:

- results in death;
- is life threatening (at the time of the event);
- requires hospitalisation or prolongation of existing inpatients' hospitalisation;
- results in persistent or significant disability or incapacity;
- is a congenital anomaly or birth defect;
- Any other important medical event that may not result in death, be life threatening, or require hospitalization, may be considered a serious adverse experience when, based upon appropriate medical judgement, the event may jeopardize the subject or may require an intervention to prevent one of the outcomes listed above.

<u>Adjustments to SAE reporting for this study:</u>

- SAE and AE will be reported until follow-up, two days after hospital discharge. Follow-up will be 15 days after cardiac surgery, at the latest. Follow-up of SAE and AE after trial phone follow-up is difficult since often these patients are treated by paediatricians or paediatric cardiologists in other hospitals. Also, patients frequently need repeated surgery or other procedures that are to be expected due to their congenital cardiac defect.
- If follow-up is not possible because parents cannot be contacted by phone, SAE and AE will be reported until 15 days after cardiac surgery.
- The above mentioned definition of the follow-up period for SAE and AE is supported by the fact that all reported SAEs and AEs so far have not been linked to trial proceedings by the DSMB and

the METC/CCMO. Also, the patient receives the trial medication in the first 48 hours after surgery and is followed for another 48 hours by pain- and sedation scores. We do not expect any adverse events to occur resulting from the trial medication 13 days after the trial medication has ended.

All SAEs will be reported to the accredited METC that approved the protocol, according to the requirements of that METC. All SAEs will be reported to the CCMO. Every center will report its own SAEs to the CCMO and to the coordinating investigator. The coordinating investigator will report the SAEs to the METC.

The sponsor will report the SAEs through the web portal *ToetsingOnline* to the accredited METC that approved the protocol, within 15 days after the sponsor has first knowledge of the serious adverse events.

SAEs that result in death or are life threatening should be reported expedited. The expedited reporting will occur not later than 7 days after the responsible investigator has first knowledge of the adverse event. This is for a preliminary report with another 8 days for completion of the report.

### 7.3 Follow-up of adverse events

All AEs will be followed until they have abated, or until a stable situation has been reached. Depending on the event, follow up may require additional tests or medical procedures as indicated, and/or referral to the general physician or a medical specialist.

SAEs need to be reported till end of study within the Netherlands, as defined in the protocol.

### 7.4 Data Safety Monitoring Board (DSMB)

A data safety board is established composed of pediatric intensivists and cardio-anesthetists with extensive clinical and research experience in the field of analgosedation on the PICU. The board consists of three doctors and a biostatistician, who also have experience in medication trials. Dr. Paola Cogo is the head of the cardiac pediatric ICU of the Bambino Gesu Hospital in Rome. Dr. Cormac Breatnach is a pediatric intensivist at the department of Paediatric Intensive Care Medicine at Our Lady's Children's Hospital, Dublin. The third member of the board and chairperson is Prof. Mr. Dr. Bas de Mol, cardiothoracic surgeon from the Medical University Hospital Amsterdam (AMC). Sten Willemsen joins the DSMB as an independent statistician. The members of the safety board have evaluated the study design during the first meeting and will receive regular updates on the status of the study. All SAE will be reported to the DSMB chairperson immediately.

A DSMB charter is drafted and includes; funding of the DSMB, a time schedule of meetings, DSMB participants and responsibilities, monitoring of the study progress, monitoring of the recruitment process, protocol violations, treatment failure, data management, number and nature of SAEs. After every review the DSMB will give recommendation regarding the appropriateness of continuing the study, from a safety prospective as well as any other recommendations relevant to the study conduct and / or patient safety.

## 8. Statistical analysis

The non-parametric Van Elteren test with stratification by center will be used to compare the primary outcome of cumulative age-adjusted morphine between groups. The secondary outcomes in the two groups will be compared using linear regression analysis with group and treatment center as categorical variables.

Analysis of secondary outcomes will include length of PICU stay, number of hours on ventilation, concomitant use of sedatives, adverse drug reactions. Adverse drug reactions will be specified in hemodynamically, gastro-intestinal, respiratory or pediatric delirium, as previously described in section two.

Analysis using robust regression models will be performed with cumulative rescue morphine (first 48 hours) as outcome variable and group (morphine vs paracetamol) as predictor variable. We will apply this method for the reason that this outcome variable is most likely non-normally distributed. Center and Down syndrome (yes/no), cyanotic vs non-cyanotic, will be added as covariates.

Any additional morphine given will be based on NRS scores of 4 and higher. In a secondary, non-inferiority analysis, we will therefore also compare the number of patients with one or more NRS scores of 4 and higher between the two groups, using a noninferiority margin of 20%. Non-inferiority will be assessed using a one-sided 97.5% confidence interval for the difference in the percentage of patients with at least one NRS score of 4 or higher between the paracetamol group and the morphine group, and non-inferiority will be proven if the upper limit of this confidence interval is lower than 20%. The confidence interval will be calculated using the method of Klingenberg (Stat Med 2014), with adjustment for center. Adverse effects and other proportions will be compared between groups by using Fisher's exact tests, and the uncertainty in these estimated proportions will be assessed using 95% confidence intervals. Level of significance will be set at 5%, and all tests will be two-sided. Non-linear mixed effect modelling (NONMEM) will be used to assess population pharmacokinetic analysis. More details are described in paragraph 6.1.3.

Morphine and its metabolites and paracetamol and its metabolites will be measured with Liquid chromatography-mass spectrometry. Non-linear mixed effect modelling (NONMEM) will be used to assess population pharmacokinetic and pharmacodynamic analysis.

## 9. Ethical considerations

### 9.1 Regulation statement

The study will be conducted to the principles of the declaration of Helsinki (version 2013) and in accordance with the Medical Research Involving Human Subjects Act (WMO).

### 9.2 Recruitment and consent

The paediatric cardiologist first informs the parents of patients who require cardiac surgery about the PACS study by means of a general flyer. The cardiologist will give parents this flyer at the outpatient clinic, several weeks before the operation. This allows parents more time to familiarize themselves with the PACS study. The flyer contains information on the PACS study, such as study aim and study procedure. The flyer informs the parents that they will approached by the researcher before the operation for study participation.  Patients who require more information are redirected to the website of the paediatric ICU of the Erasmus MC – Sophia children's hospital. This website contains more detailed information on the PACS study as well as background information on the ward and the experience of the ICU with pain and sedation trials. The official patient information will be available through a link on this website.

Patients are admitted to the general ward the day before the cardiac surgery. The parents of the patient and/or his legal representatives will then in person be informed about the study by the investigator. It is not possible to involve the treating physician in the informed consent procedure, since there are several specialisms involved in the care of these children. The paediatric cardiologist takes care of the children in the general ward, but during the PACS trial the child will mostly be in the care of the cardiac surgeon, the paediatric cardio-anaesthesiologist and the paediatric intensivist.  For the sake of continuity the informed consent procedure will be done by dedicated researchers.

Parents of the patient and/or the legal representatives, have until the next morning after talking to the researcher to consider participating in the PACS study. Parents hwo have read the general flyer from the cardiologist will have had more time to consider participating in the PACS study. They will also have had the opportunity to gather more background information on the trial and the PICU of the Erasmus MC-Sophia children's hospital.

### 9.3 Objection by minors or incapacitated subjects (if applicable)

Sampling will be done from the arterial line which all patients have after surgery. Since this is not an invasive procedure, we do not expect any resistance from the child.

### 9.4 Benefit and risks assessment, group relatedness

The risks and burdens associated with this study are negligible. Blood samples are only taken from an indwelling arterial catheter, which is already in place for clinical purposes). Sedation and pain scores are observational procedures already performed for clinical purposes. Possible burden and risk of participating in the study is the risk of insufficient analgesia after cardiac surgery with intermittent

intravenous paracetamol. Given previous studies on the postoperative efficacy of paracetamol in children after major non cardiac surgery, this risk is considered low. Moreover, morphine will be administered as rescue medication in case of insufficient analgesia in both groups.

This research is group-related as data for healthy adults or healthy children cannot be extrapolated to these hemodynamically compromised, critically ill children who need the medication prescribed.

Standard therapy for the relief of pain in these children is normally performed through the administration of morphine, which has several adverse drug reactions especially in the patient group described in this protocol. To reduce the adverse reactions it is necessary to have an alternative (co-)medicine for the use of pain relief. Intravenous paracetamol is potentially the replacement or additive analgesic drug for morphine and may therefore reduce the occurrence of morphine related adverse drug reactions. Previous studies showed analgesic effects of paracetamol, when administered intravenously in children undergoing major non-cardiac surgery. Moreover, all patients in this study receive a loading dose of 100 ug/kg morphine at the end of surgery and rescue doses are morphine are foreseen according to a strict analgesic protocol (appendix 4)

### 9.5 Compensation for injury

The sponsor/investigator has a liability insurance which is in accordance with article 7 of the WMO.

The sponsor (also) has an insurance which is in accordance with the legal requirements in the Netherlands (Article 7 WMO). This insurance provides cover for damage to research subjects through injury or death caused by the study.

The insurance applies to the damage that becomes apparent during the study or within 4 years after the end of the study.

## 10. Administrative aspects and publication

### 10.1 Handling and storage of data and documents

Data and blood samples will be stored for 15 years, using a subject's identification code list, of which the key will be accessible to the researcher. The investigator will arrange for retention of the subjects identification code list for at least discontinuation of the trial. Subjects' files will be kept for the maximum period of time permitted by the center. Other documentation belonging to the trial will be archived for 15 years in the investigator file.

### 10.2 Monitoring and Quality Assurance

Monitoring will take place in accordance with the monitoring plan in appendix 10.

### 10.3 Amendments

Amendments are changes made to the research after favourable opinion by the accredited METC has been given. All amendments will be notified to the METC that gave a favourable opinion.

### 10.4 Annual progress report

The investigator will submit a summary of the progress of the trial to the accredited METC once a year. Information will be provided on the date of inclusion of the first subject, numbers of subjects included and numbers of subjects that have completes the trial, serious adverse events, other problems and amendments.

### 10.5 End of study report

The investigator will notify the accredited METC of the end of the study within a period of 8 weeks. The end of the study is defined as the last patient's last visit.

In case the study is ended prematurely, the investigator will notify the accredited METC within 15 days, including the reasons for the premature termination.

Within one year after the end of the study, the investigator/sponsor will submit a final study report with the results of the study, including any publications/abstracts of the study, to the accredited METC.

PACS study; Pediatric Analgesia after Cardiac Surgery.

## 11. References


1. van der Linde D, Konings EE, Slager MA, Witsenburg M, Helbing WA, Takkenberg JJ, et al. Birth prevalence of congenital heart disease worldwide: a systematic review and meta-analysis. J Am Coll Cardiol. 2011;58(21):2241-7.
2. Dolk H, Loane M, Garne E, European Surveillance of Congenital Anomalies Working G. Congenital heart defects in Europe: prevalence and perinatal mortality, 2000 to 2005. Circulation. 2011;123(8):841-9.
3. Nederlandse Vereniging voor Thoraxchirurgie. Aantallen en uitkomsten van congenitale cardiothoracale chirurgie in Nederland [website]. [Available from: http://www.nvtnet.nl/index.asp?page_id=129.
4. Anand KJ, Aynsley-Green A. Measuring the severity of surgical stress in newborn infants. J Pediatr Surg. 1988;23(4):297-305.
5. Taddio A, Katz J, Ilersich AL, Koren G. Effect of neonatal circumcision on pain response during subsequent routine vaccination. Lancet. 1997;349(9052):599-603.
6. Weisman SJ, Bernstein B, Schechter NL. Consequences of inadequate analgesia during painful procedures in children. Arch Pediatr Adolesc Med. 1998;152(2):147-9.
7. Berde CB, Jaksic T, Lynn AM, Maxwell LG, Soriano SG, Tibboel D. Anesthesia and analgesia during and after surgery in neonates. Clin Ther. 2005;27(6):900-21.
8. Hammer GB, Golianu B. Opioid analgesia in neonates following cardiac surgery. Semin Cardiothorac Vasc Anesth. 2007;11(1):47-58.
9. van Dijk M, Bouwmeester NJ, Duivenvoorden HJ, Koot HM, Tibboel D, Passchier J, et al. Efficacy of continuous versus intermittent morphine administration after major surgery in 0-3-year-old infants; a double-blind randomized controlled trial. Pain. 2002;98(3):305-13.
10. Knibbe CA, Krekels EH, van den Anker JN, DeJongh J, Santen GW, van Dijk M, et al. Morphine glucuronidation in preterm neonates, infants and children younger than 3 years. Clin Pharmacokinet. 2009;48(6):371-85.
11. Krekels EHJ, Tibboel D, Danhof M, Knibbe CAJ. Prediction of Morphine Clearance in the Paediatric Population: How Accurate are the Available Pharmacokinetic Models? Clin Pharmacokinet. 2012;51:698-700.
12. de Wildt SN, Kearns GL, Leeder JS, van den Anker JN. Glucuronidation in humans. Pharmacogenetic and developmental aspects. Clin Pharmacokinet. 1999;36(6):439-52.
13. Kearns GL, Abdel-Rahman SM, Alander SW, Blowey DL, Leeder JS, Kauffman RE. Developmental pharmacology--drug disposition, action, and therapy in infants and children. N Engl J Med. 2003;349(12):1157-67.
14. Krekels EH, DeJongh J, van Lingen RA, van der Marel CD, Choonara I, Lynn AM, et al. Predictive performance of a recently developed population pharmacokinetic model for morphine and its metabolites in new datasets of (preterm) neonates, infants and children. Clin Pharmacokinet. 2011;50(1):51-63.
15. Krekels EH, Tibboel D, de Wildt SN, Ceelie I, Dahan A, van Dijk M, et al. Evidence-based morphine dosing for postoperative neonates and infants. Clin Pharmacokinet. 2014;53(6):553-63.
16. Ilse Ceelie, Saskia N. de Wildt, Monique van Dijk, Margreeth M. J. van den Berg, Gerbrich E. van den Bosch, Hugo J. Duivenvoorden, et al. Effect of Intravenous Paracetamol on Postoperative Morphine Requirements in Neonates and Infants Undergoing Major Noncardiac Surgery. JAMA. 2013;309(2):149-54.
17. Trescot AM. Genetics and implications in perioperative analgesia. Best Pract Res Clin Anaesthesiol. 2014;28(2):153-66.

PACS study; Pediatric Analgesia after Cardiac Surgery.


18. Smith MT, Muralidharan A. Pharmacogenetics of pain and analgesia. Clin Genet. 2012;82(4):321-30.
19. Association of Paediatric Anaesthetists of Great B, Ireland. Good practice in postoperative and procedural pain management, 2nd edition. Paediatr Anaesth. 2012;22 Suppl 1:1-79.
20. Howard RF, Lloyd-Thomas A, Thomas M, Williams DG, Saul R, Bruce E, et al. Nurse-controlled analgesia (NCA) following major surgery in 10,000 patients in a children's hospital. Paediatr Anaesth. 2010;20(2):126-34.
21. Lynn AM, Nespeca MK, Opheim KE, Slattery JT. Respiratory effects of intravenous morphine infusions in neonates, infants, and children after cardiac surgery. ANESTH ANALG. 1993;77(4):695-701.
22. Dagan O, Klein J, Bohn D, Barker G, Koren G. Morphine pharmacokinetics in children following cardiac surgery: Effects of disease and inotropic support. J CARDIOTHORAC VASC ANESTH. 1993;7(4):396-8.
23. Ahlers SJGM. Pain still hurts : pain assessment and pain management in intensive care patients [Thesis]. Rotterdam: Erasmus University; 2012.
24. Lotsch J, Skarke C, Schmidt H, Liefhold J, Geisslinger G. Pharmacokinetic modeling to predict morphine and morphine-6-glucuronide plasma concentrations in healthy young volunteers. Clin Pharmacol Ther. 2002;72(2):151-62.
25. van Saet A, de Wildt SN, Knibbe CA, Bogers AD, Stolker RJ, Tibboel D. The effect of adult and pediatric cardiopulmonary bypass on pharmacokinetic and pharmacodynamic parameters. Curr Clin Pharmacol. 2013;8(4):297-318.
26. Anne Lynn, Mary Kay Nespeca, Susan L. Bratton, Susan G. Strauss, Shen DD. Clearance of Morphine in Postoperative Infants During Intravenous Infusion: The Influence of Age and Surgery. Anesth Analg. 1998;86:958-63.
27. van den Broek MP, Groenendaal F, Egberts AC, Rademaker CM. Effects of hypothermia on pharmacokinetics and pharmacodynamics: a systematic review of preclinical and clinical studies. Clin Pharmacokinet. 2010;49(5):277-94.
28. Tortorici MA, Kochanek PM, Poloyac SM. Effects of hypothermia on drug disposition, metabolism, and response: A focus of hypothermia-mediated alterations on the cytochrome P450 enzyme system. Crit Care Med. 2007;35(9):2196-204.
29. Bjelland TW, Klepstad P, Haugen BO, Nilsen T, Dale O. Effects of hypothermia on the disposition of morphine, midazolam, fentanyl, and propofol in intensive care unit patients. Drug Metab Dispos. 2013;41(1):214-23.
30. Wildschut ED, van Saet A, Pokorna P, Ahsman MJ, Van den Anker JN, Tibboel D. The Impact of Extracorporeal Life Support and Hypothermia on Drug Disposition in Critically Ill Infants and Children. Pediatr Clin North Am. 2012;59(5):1184-204.
31. Chan JK, Roth J, Oppenheim JJ, Tracey KJ, Vogl T, Feldmann M, et al. Alarmins: awaiting a clinical response. J Clin Invest. 2012;122(8):2711-9.
32. Manson J, Thiemermann C, Brohi K. Trauma alarmins as activators of damage-induced inflammation. Br J Surg. 2012;99 Suppl 1:12-20.
33. Durandy Y. Minimizing systemic inflammation during cardiopulmonary bypass in the pediatric population. Artif Organs. 2014;38(1):11-8.
34. Hall RI. Cardiopulmonary bypass and the systemic inflammatory response: effects on drug action. J Cardiothorac Vasc Anesth. 2002;16(1):83-98.
35. Damgaard S, Nielsen CH, Andersen LW, Bendtzen K, Tvede M, Steinbruchel DA. Cell saver for on-pump coronary operations reduces systemic inflammatory markers: a randomized trial. ANN THORAC SURG. 2010;89(5):1511-7.

PACS study; Pediatric Analgesia after Cardiac Surgery.

36. Gessler P, Hohl V, Carrel T, Pfenninger J, Schmid ER, Baenziger O, et al. Administration of steroids in pediatric cardiac surgery: impact on clinical outcome and systemic inflammatory response. Pediatr Cardiol. 2005;26(5):595-600.
37. Dieleman JM, Nierich AP, Rosseel PM, van der Maaten JM, Hofland J, Diephuis JC, et al. Intraoperative high-dose dexamethasone for cardiac surgery: a randomized controlled trial. JAMA. 2012;308(17):1761-7.
38. Keski-Nisula J, Pesonen E, Olkkola KT, Peltola K, Neuvonen PJ, Tuominen N, et al. Methylprednisolone in neonatal cardiac surgery: reduced inflammation without improved clinical outcome. ANN THORAC SURG. 2013;95(6):2126-32.
39. de Jong PR, Schadenberg AW, van den Broek T, Beekman JM, van Wijk F, Coffer PJ, et al. STAT3 regulates monocyte TNF-alpha production in systemic inflammation caused by cardiac surgery with cardiopulmonary bypass. PLoS ONE. 2012;7(4):e35070.
40. Dawson PJ, Bjorksten AR, Blake DW, Goldblatt JC. The effects of cardiopulmonary bypass on total and unbound plasma concentrations of propofol and midazolam. J CARDIOTHORAC VASC ANESTH. 1997;11(5):556-61.
41. Rosen DA, Rosen KR, Silvasi DL. In vitro variability in fentanyl absorption by different membrane oxygenators. J Cardiothorac Anesth. 1990;4(3):332-5.
42. Myers GJ, Voorhees C, Eke B, Johnstone R. The effect of Diprivan (propofol) on phosphorylcholine surfaces during cardiopulmonary bypass — an in vitro investigation. Perfusion. 2009;24(5):349-55.
43. Wolf AR, Jackman L. Analgesia and sedation after pediatric cardiac surgery. Paediatr Anaesth. 2011;21(5):567-76.
44. Harizanov R, Rainova I, Tzvetkova N, Kaftandjiev I, Bikov I, Mikov O. Geographical distribution and epidemiological characteristics of visceral leishmaniasis in Bulgaria, 1988 to 2012. Euro Surveill. 2013;18(29):20531.
45. Algra SO, Jansen NJ, van der Tweel I, Schouten AN, Groenendaal F, Toet M, et al. Neurological injury after neonatal cardiac surgery: a randomized, controlled trial of 2 perfusion techniques. Circulation. 2014;129(2):224-33.
46. O'Brien VP, Bokelmann K, Ramirez J, Jobst K, Ratain MJ, Brockmoller J, et al. Hepatocyte nuclear factor 1 regulates the expression of the organic cation transporter 1 via binding to an evolutionary conserved region in intron 1 of the OCT1 gene. J Pharmacol Exp Ther. 2013;347(1):181-92.
47. Dimitropoulos A, McQuillen PS, Sethi V, Moosa A, Chau V, Xu D, et al. Brain injury and development in newborns with critical congenital heart disease. Neurology. 2013;81(3):241-8.
48. Brown WR, Moody DM, Challa VR, Stump DA, Hammon JW. Longer duration of cardiopulmonary bypass is associated with greater numbers of cerebral microemboli. Stroke. 2000;31(3):707-13.
49. Fudulu DP, Gibbison B, Upton T, Stoica SC, Caputo M, Lightman S, et al. Corticosteroids in Pediatric Heart Surgery: Myth or Reality. Frontiers in Pediatrics. 2018;6:112.
50. Van den Berghe G. On the Neuroendocrinopathy of Critical Illness. Perspectives for Feeding and Novel Treatments. Am J Respir Crit Care Med. 2016;194(11):1337-48.
51. Boonen E, Vervenne H, Meersseman P, Andrew R, Mortier L, Declercq PE, et al. Reduced cortisol metabolism during critical illness. N Engl J Med. 2013;368(16):1477-88.
52. Vuong C, Van Uum SH, O'Dell LE, Lutfy K, Friedman TC. The effects of opioids and opioid analogs on animal and human endocrine systems. Endocr Rev. 2010;31(1):98-132.
53. Peeters B, Guiza F, Boonen E, Meersseman P, Langouche L, Van den Berghe G. Drug-induced HPA axis alterations during acute critical illness: a multivariable association study. Clin Endocrinol (Oxf). 2017;86(1):26-36.
54. Sabatino G, Quartulli L, Di Fabio S, Ramenghi LA. Hemodynamic effects of intravenous morphine infusion in ventilated preterm babies. Early Hum Dev. 1997;47(3):263-70.

PACS study; Pediatric Analgesia after Cardiac Surgery.

55. Heinle JS, Diaz LK, Fox LS. Early extubation after cardiac operations in neonates and young infants. J Thorac Cardiovasc Surg. 1997;114(3):413-8.
56. Ivan Wong, Celia St John-Green, Walker SM. Opioid-sparing effects of perioperative paracetamol and nonsteroidal anti-inflammatory drugs (NSAIDs) in children. Pediatric Anesthesia. 2013;23:475-95.
57. Kumpulainen E, Kokki H, Halonen T, Heikkinen M, Savolainen J, Laisalmi M. Paracetamol (acetaminophen) penetrates readily into the cerebrospinal fluid of children after intravenous administration. Pediatrics. 2007;119(4):766-71.
58. Pickering G, Loriot MA, Libert F, Eschalier A, Beaune P, Dubray C. Analgesic effect of acetaminophen in humans: first evidence of a central serotonergic mechanism. Clin Pharmacol Ther. 2006;79(4):371-8.
59. Lizi Zhao, Pickering G. Paracetamol metabolism and related genetic differences. Drug Metabolism Reviews. 2011;43(1):41-52.
60. McGill MR, Jaeschke H. Metabolism and disposition of acetaminophen: recent advances in relation to hepatotoxicity and diagnosis. Pharm Res. 2013;30(9):2174-87.
61. van der Marel CD, Anderson BJ, van Lingen RA, Holford NH, Pluim MA, Jansman FG, et al. Paracetamol and metabolite pharmacokinetics in infants. Eur J Clin Pharmacol. 2003;59(3):243-51.
62. Brian J. Anderson, Gerard Pons, Elisabeth Autret-Leca, Karel Allegaert, Boccard E. Pediatric intravenous paracetamol (propacetamol) pharmacokinetics: a population analysis. Pediatric Anesthesia. 2005;15:282-92.
63. Beasley R, Clayton T, Crane J, von Mutius E, Lai CK, Montefort S, et al. Association between paracetamol use in infancy and childhood, and risk of asthma, rhinoconjunctivitis, and eczema in children aged 6-7 years: analysis from Phase Three of the ISAAC programme. Lancet. 2008;372(9643):1039-48.
64. Heintze K, Petersen KU. The case of drug causation of childhood asthma: antibiotics and paracetamol. Eur J Clin Pharmacol. 2013;69(6):1197-209.
65. van der Marel CD, van Lingen RA, Pluim MA, Scoones G, van Dijk M, Vaandrager JM, et al. Analgesic efficacy of rectal versus oral acetaminophen in children after major craniofacial surgery. Clin Pharmacol Ther. 2001;70(1):82-90.
66. van der Marel CD, Peters JWB, Bouwmeester NJ, Jacqz-Aigrain E, van den Anker JN, Tibboel D. Rectal acetaminophen does not reduce morphine consumption after major surgery in young infants. Br J Anaesth. 2007;98(3):372-9.
67. Moller PL, Juhl GI, Payen-Champenois C, Skoglund LA. Intravenous acetaminophen (paracetamol): comparable analgesic efficacy, but better local safety than its prodrug, propacetamol, for postoperative pain after third molar surgery. ANESTH ANALG. 2005;101(1):90-6, table of contents.
68. Walson PD, Jones J, Chesney R, Rodarte A. Antipyretic efficacy and tolerability of a single intravenous dose of the acetaminophen prodrug propacetamol in children: a randomized, double-blind, placebo-controlled trial. Clin Ther. 2006;28(5):762-9.
69. B. S. Mohammed, T. Engelhardt, G. A. Cameron, L. Cameron, G. M. Hawksworth, A. F. Hawwa, et al. Population pharmacokinetics of single-dose intravenous paracetamol in children. British Journal of Anasthesia. 2012;108(5):823-9.
70. Wang C, Sadhavisvam S, Krekels EH, Dahan A, Tibboel D, Danhof M, et al. Developmental changes in morphine clearance across the entire paediatric age range are best described by a bodyweight-dependent exponent model. Clin Drug Investig. 2013;33(7):523-34.
71. Ren ZY, Xu XQ, Bao YP, He J, Shi L, Deng JH, et al. The impact of genetic variation on sensitivity to opioid analgesics in patients with postoperative pain: a systematic review and meta-analysis. Pain Physician. 2015;18(2):131-52.
72. Trescot AM, Faynboym S. A review of the role of genetic testing in pain medicine. Pain Physician. 2014;17(5):425-45.

73. Krasniak AE, Knipp GT, Svensson CK, Liu W. Pharmacogenomics of acetaminophen in pediatric populations: a moving target. Front Genet. 2014;5:314.
74. Darbari DS, van Schaik RH, Capparelli EV, Rana S, McCarter R, van den Anker J. UGT2B7 promoter variant -840G>A contributes to the variability in hepatic clearance of morphine in patients with sickle cell disease. Am J Hematol. 2008;83(3):200-2.
75. Matic M, Norman E, Rane A, Beck O, Andersson M, Elens L, et al. Effect of UGT2B7 -900G>A (-842G>A; rs7438135) on morphine glucuronidation in preterm newborns: results from a pilot cohort. Pharmacogenomics. 2014;15(12):1589-97.
76. Fukuda T, Chidambaran V, Mizuno T, Venkatasubramanian R, Ngamprasertwong P, Olbrecht V, et al. OCT1 genetic variants influence the pharmacokinetics of morphine in children. Pharmacogenomics. 2013;14(10):1141-51.
77. Venkatasubramanian R, Fukuda T, Niu J, Mizuno T, Chidambaran V, Vinks AA, et al. ABCC3 and OCT1 genotypes influence pharmacokinetics of morphine in children. Pharmacogenomics. 2014;15(10):1297-309.
78. Tzvetkov MV, dos Santos Pereira JN, Meineke I, Saadatmand AR, Stingl JC, Brockmoller J. Morphine is a substrate of the organic cation transporter OCT1 and polymorphisms in OCT1 gene affect morphine pharmacokinetics after codeine administration. Biochem Pharmacol. 2013;86(5):666-78.
79. Court MH, Peter I, Hazarika S, Vasiadi M, Greenblatt DJ, Lee WM, et al. Candidate gene polymorphisms in patients with acetaminophen-induced acute liver failure. Drug Metab Dispos. 2014;42(1):28-32.
80. Ueshima Y, Tsutsumi M, Takase S, Matsuda Y, Kawahara H. Acetaminophen metabolism in patients with different cytochrome P-4502E1 genotypes. Alcohol Clin Exp Res. 1996;20(1 Suppl):25A-8A.
81. Valkenburg AJ. Without Uttering a Word: Pain assessment and management in intellectually disabled children [Thesis]. Rotterdam: Erasmus University; 2012.
82. Lynn A, Nespeca MK, Bratton SL, Strauss SG, Shen DD. Clearance of morphine in postoperative infants during intravenous infusion: The influence of age and surgery. Anesth Analg. 1998;86(5):958-63.
83. Berkenstadt H, Segal E, Mayan H, Almog S, Rotenberg M, Perel A, et al. The pharmacokinetics of morphine and lidocaine in critically ill patients. Intensive Care Med. 1999;25(1):110-2.
84. Algra SO, Schouten AN, Jansen NJ, van Oeveren W, Haas F, Groenendaal F, et al. Perioperative and bedside cerebral monitoring identifies cerebral injury after surgical correction of congenital aortic arch obstruction. Intensive Care Med. 2015.
85. Schadenberg AW, van den Broek T, Siemelink MA, Algra SO, de Jong PR, Jansen NJ, et al. Differential homeostatic dynamics of human regulatory T-cell subsets following neonatal thymectomy. J Allergy Clin Immunol. 2014;133(1):277-80 e1-6.
86. Algra SO, Driessen MM, Schadenberg AW, Schouten AN, Haas F, Bollen CW, et al. Bedside prediction rule for infections after pediatric cardiac surgery. Intensive Care Med. 2012;38(3):474-81.
87. Algra SO, Groeneveld KM, Schadenberg AW, Haas F, Evens FC, Meerding J, et al. Cerebral ischemia initiates an immediate innate immune response in neonates during cardiac surgery. J Neuroinflammation. 2013;10:24.
88. van Wijk F, Prakken B. Heat shock proteins: Darwinistic immune modulation on dangerous grounds. J Leukoc Biol. 2010;88(3):431-4.
89. Bobek D, Grcevic D, Kovacic N, Lukic IK, Jelusic M. The presence of high mobility group box-1 and soluble receptor for advanced glycation end-products in juvenile idiopathic arthritis and juvenile systemic lupus erythematosus. Pediatr Rheumatol Online J. 2014;12:50.
90. Franck LS, Ridout D, Howard R, Peters J, Honour JW. A comparison of pain measures in newborn infants after cardiac surgery. Pain. 2011.

91. Valkenburg AJ, Boerlage AA, Ista E, Duivenvoorden HJ, Tibboel D, van Dijk M. The COMFORT-behavior scale is useful to assess pain and distress in 0- to 3-year-old children with Down syndrome. Pain. 2011;152(9):2059-64.
92. Monique van Dijk, Josien B. de Boer, Hans M. Koot, Dick Tibboel, Jan Passchier, Duivenvoorden HJ. The reliability and validity of the COMFORT scale as a postoperative pain instrument in 0 to 3-year-old infants. Pain. 2000;84:367-77.
93. Boerlage AA, Ista E, Duivenvoorden HJ, de Wildt SN, Tibboel D, van Dijk M. The COMFORT behaviour scale detects clinically meaningful effects of analgesic and sedative treatment. Eur J Pain. 2014.
94. Ista E, van Dijk M, Tibboel D, de Hoog M. Assessment of sedation levels in pediatric intensive care patients can be improved by using the COMFORT "behavior" scale. Pediatr Crit Care Med. 2005;6(1):58-63.
95. von Baeyer CL, Chambers CT, Eakins DM. Development of a 10-item short form of the parents' postoperative pain measure: the PPPM-SF. J Pain. 2011;12(3):401-6.
96. Ista E, van Dijk M, Gischler S, de Leeuw M, Poley MJ, Tibboel D. Weaning of opioids and benzodiazepines at home after critical illness in infants: a cost-effective approach. J Opioid Manag. 2010;6(1):55-62.
97. Ista E, van Dijk M, de Hoog M, Tibboel D, Duivenvoorden HJ. Construction of the Sophia Observation withdrawal Symptoms-scale (SOS) for critically ill children. Intensive Care Med. 2009;35(6):1075-81.
98. Pollack MM, Patel KM, Ruttimann UE. PRISM III: an updated Pediatric Risk of Mortality score. Crit Care Med. 1996;24(5):743-52.
99. Larsen SH, Pedersen J, Jacobsen J, Johnsen SP, Hansen OK, Hjortdal V. The RACHS-1 risk categories reflect mortality and length of stay in a Danish population of children operated for congenital heart disease. Eur J Cardiothorac Surg. 2005;28(6):877-81.
100. Bojan M, Gerelli S, Gioanni S, Pouard P, Vouhe P. The Aristotle Comprehensive Complexity score predicts mortality and morbidity after congenital heart surgery. ANN THORAC SURG. 2011;91(4):1214-21.
101. Bojan M, Gerelli S, Gioanni S, Pouard P, Vouhe P. Comparative study of the Aristotle Comprehensive Complexity and the Risk Adjustment in Congenital Heart Surgery scores. ANN THORAC SURG. 2011;92(3):949-56.
102. Leteurtre S, Duhamel A, Salleron J, Grandbastien B, Lacroix J, Leclerc F, et al. PELOD-2: an update of the PEdiatric logistic organ dysfunction score. Crit Care Med. 2013;41(7):1761-73.
103. Valkenburg AJ, van Dijk M, de Leeuw TG, Meeussen CJ, Knibbe CA, Tibboel D. Anaesthesia and postoperative analgesia in surgical neonates with or without Down's syndrome: is it really different? BR J ANAESTH. 2012;108(2):295-301.

## Appendix 1: summary of results worldwide survey

**Table 1: summary of analgesic drugs prescribed to children after cardiac surgery.**

| Age groups | NL | EU | Non-EU |
|---|---|---|---|
| Neonates<br>0-28 days | - morphine IV bolus 100 mcg/kg<br>- morphine continue IV 10-40 mcg/kg/h<br>- fentanyl 1-5 mcg/kg/h<br>- paracetamol IV 7.5 mg/kg Q8h<br>- paracetamol PR 60-90 mg/kg/day | - morphine IV bolus 50-200 mcg/kg<br>- morphine IV 10-30 mcg/kg/h<br>- piritramide IV bolus 0.2 mg (<15kg)<br>- piritramide 1.2 mg/kg/day<br>- fentanyl IV bolus 1-2 mcg/kg<br>- fentanyl IV 2-6 mcg/kg/h<br>- remifentanil IV bolus 1 mcg/kg<br>- remifentanil IV 0.1-0.2 mcg/kg/min<br>- sufentanil 1- 2 mcg/kg/h<br>- paracetamol IV 7.5 mg/kg Q6h<br>- paracetamol PR 15-20 mg/kg Q8h<br>- metamizol IV 40 mg/kg<br>- dexketoprofen IV 0.5-1 mg/kg | - morphine IV bolus 50-100 mcg/kg<br>- morphine IV 5-20 mcg/kg/h |
| Infants<br>29 days – 3 years | - morphine bolus IV 100 mcg/kg<br>- morphine continue IV 10-40 mcg/kg/h<br>- fentanyl 1-5 mcg/kg/h<br>- paracetamol IV 7.5-15 mg/kg Q6h<br>- paracetamol PR 60-90 mg/kg/day<br>- diclofenac (>3 months)2-3 mg/kg/day | - morphine IV bolus 50-500 mcg/kg,<br>- morphine IV 20-60 mcg/kg/h<br>- piritramide IV bolus 0.05-0.4 mg/kg<br>- piritramide IV 1.2 mg/kg/day<br>- fentanyl IV bolus 1-2 mcg/kg<br>- fentanyl IV 2-6 mcg/kg/h<br>- remifentanil IV bolus 1 mcg/kg<br>- remifentanil IV 0.1-0.2 mcg/kg/min<br>- sufentanil 1- 2 mcg/kg/h<br>- dexmedetomidin 0,5 mcg/kg/h<br>- paracetamol IV. 7.5-15 mg/kg Q6h<br>- paracetamol PR 15mg/kg Q8h<br>- diclofenac (> 6 months) PR 1mg/kg Q8h<br>- ibuprofen PO bolus 5-10 mg/kg<br>- dexketoprofen 0.5-1 mg/kg<br>- metamizol 40 mg/kg | - morphine IV bolus 50-100 mcg/kg<br>- morphine IV 10-40 mcg/kg/day<br>- dexmedotomidine IV bolus 50 mcg/kg<br>- dexmedotomide IV 0.5-1.5 mcg/kg/min<br>- paracetamol PO 15 mg/kg, Q6h |
| Pain and sedation assessment method | - COMFORT-B<br>- NRS<br>- VASobs<br>- vital parameters and clinical observation nurse and doctor | - COMFORT-B<br>- NRS<br>- VASobs<br>- BSS<br>- FLACC<br>- LLANTO SCALE | - COMFORT-B<br>- Bedsite assessment of nurse and doctor |

**Results of a self-reported survey. Participants: Erasmus MC-sophia, Rotterdam; LUMC, Leiden; UMC Utrecht; UMC Groningen; Our Lady's Childrens Hospital, Crumlin; Childrens Hospital Bambino Gesu, Rome; Royal Brompton Hospital, London; Royal Children's Hopital, Melbourne; University Hospital, Leuven; University Hospital La Paz, Madrid; Starship Childrens Hospital, Auckland; Hospital for sick children, Toronto; German Heart Center, Munich; Queen Silvia Hospital Gothenburg, Memorial Hospital – Child Health Center, Warschau.**

## Appendix 2: morphine dosing regimen

All patients will receive a loading dose of morphine at the end of surgery. The loading dose will be 100 mcg/kg morphine. In hemodynamically unstable patients this loading dose can be given in two hours, preferably the total dose will remain 100 mcg/kg/h.
After the loading dose patients will receive the study medication. Paracetamol dosing will be according to Dutch guidelines. Morphine dosing will be based on data from cardiac and non-cardiac surgery patients aged 0-3 years (81, 103). This data comes from participants in previous trials (11, 15, 16, 70).

Morphine:
Continuous infusion:

| Weight | Infusion rate μg/kg/h | Infusion rate μg/h |
|---|---|---|
| 0.5 – 1 | 1.6 | 1.2 |
| 1 – 1.5 | 2.1 | 2.6 |
| 1.5 – 2 | 2.5 | 4.4 |
| 2 – 2.5 | 3.1 | 6.9 |
| 2.5 – 3 | 3.9 | 10.8 |
| 3 – 3.5 | 5.3 | 17.1 |
| 3.5 – 4 | 7.1 | 26.7 |
| 4 – 4.5 | 9.3 | 39.4 |
| 4.5 – 5 | 11.4 | 54.1 |
| 5 – 5.5 | 13.2 | 69.1 |
| 5.5 – 6 | 14.5 | 83.3 |
| 6 – 6.5 | 15.4 | 96.3 |
| 6.5 – 7 | 16.0 | 108 |
| 7 – 7.5 | 16.4 | 119 |
| 7.5 – 8 | 16.6 | 129 |
| 8 – 9 | 16.8 | 143 |
| 9 – 10 | 16.9 | 160 |
| 10 – 11 | 16.8 | 177 |
| 11 – 12 | 16.7 | 192 |
| 12 – 13 | 16.6 | 208 |
| 13 – 14 | 16.5 | 222 |
| 14 – 15 | 16.4 | 237 |
| >15 | 16.0 | - |

Paracetamol:
37 weeks PNA – 1 month:
- loading dose 20 mg/kg
- intermittent infusion 40 mg/kg/day over Q6 hours

1 month – 18 years:
- loading dose 20 mg/kg, maximum 1 gram/dose
- intermittent infusion 60 mg/kg/day over Q6 hours, maximum 4 gram/day, max dose 1 gram/dose

## Appendix 3: sedation protocol (page 1)

### Bepaling keuze sedatie of pijn algoritme

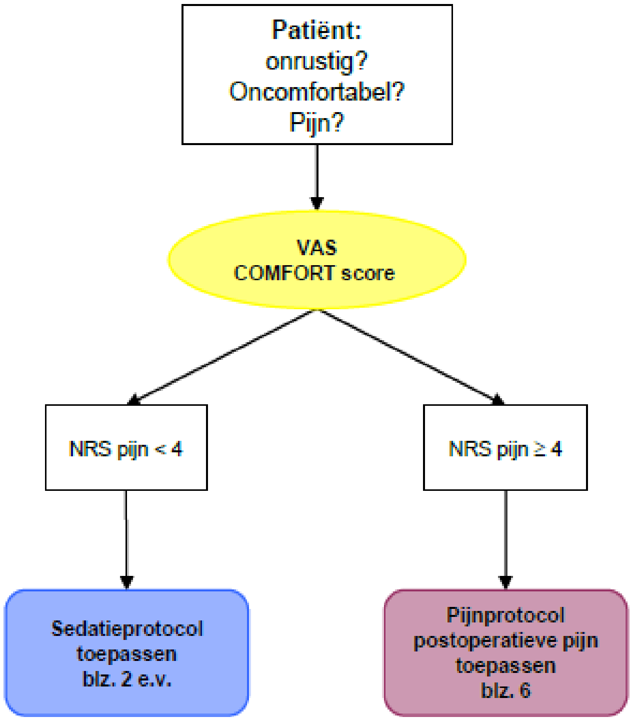


### Afkappunten sedatieprotocol *

| Afkappunten sedatie COMFORT gedragschaal | | |
|---|---|---|
| 6 - 10 | 11 - 22<br>grijs gebied | 23 - 30 |
| Overweeg sedativa af te bouwen | Verpleegkundige mening vereist Verpleegkundige Interpretatie Sedatie Score (VISS)<br><br>**Sedatie is (VISS):** **Medicatie:**<br>Onvoldoende → verhogen<br>Adequaat → zo laten<br>Teveel → verlagen | Overweeg sedativa te verhogen |

* Ista E. et al. Assessment of sedation levels in pediatric intensive care patients can be improved by using the COMFORT 'behavior' scale. Ped Crit Care Med 2005; 6:58-63

**Appendix 3: sedation protocol (page 2)**

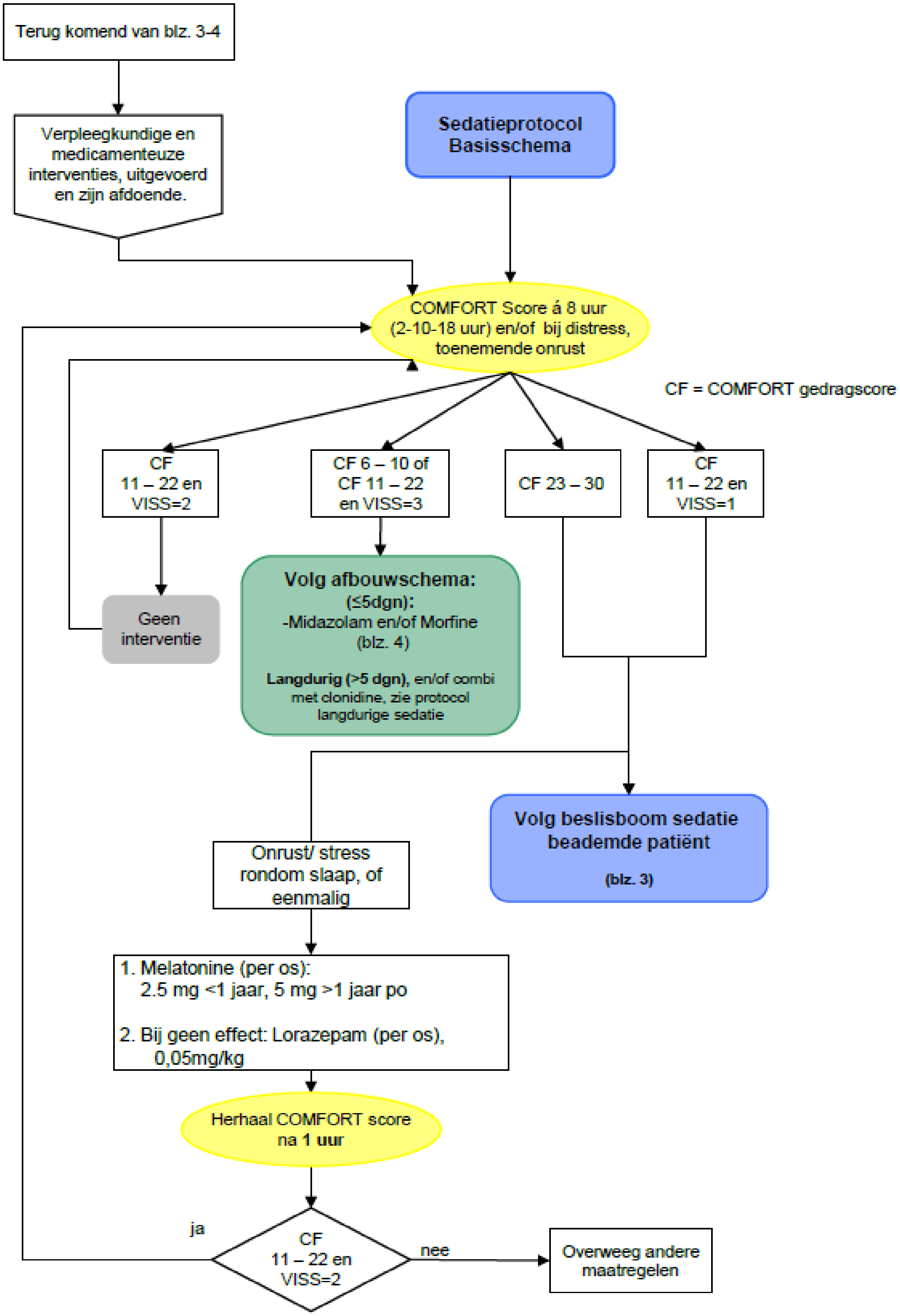

**Appendix 3: sedation protocol (page 3)**

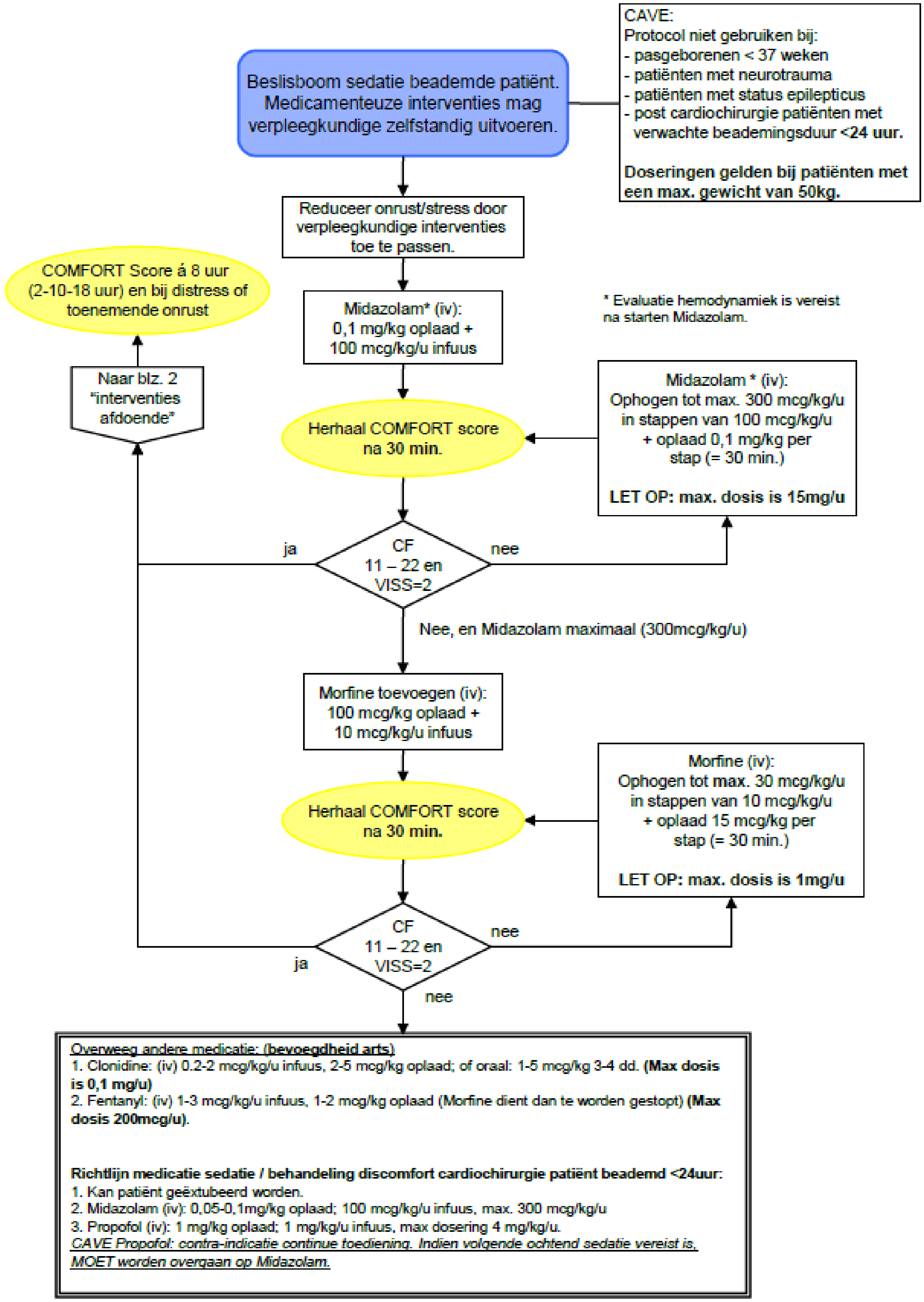

**Appendix 3: sedation protocol (page 4)**

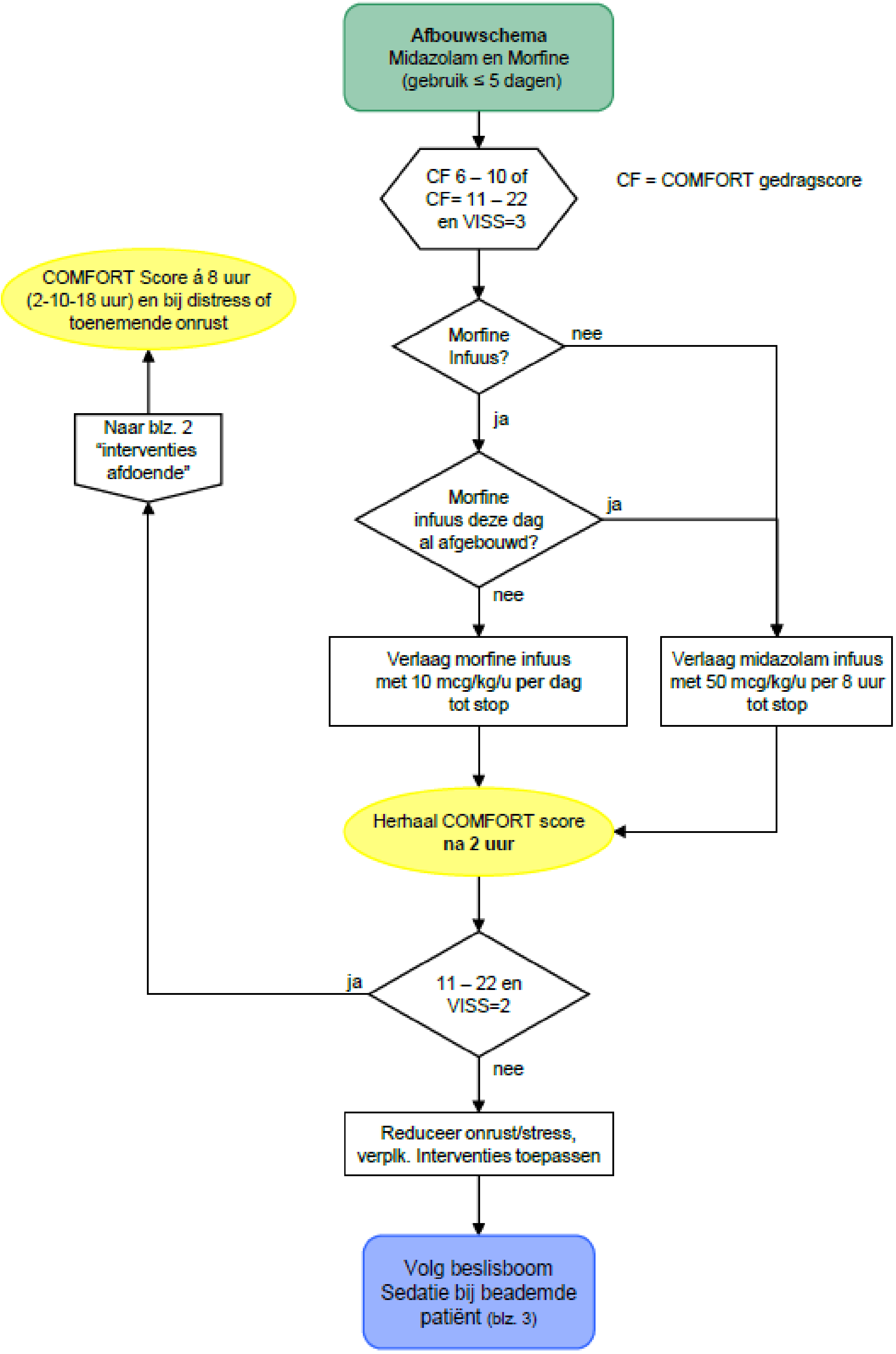

## Appendix 4: post-operative pain protocol in children 0-3 years of age

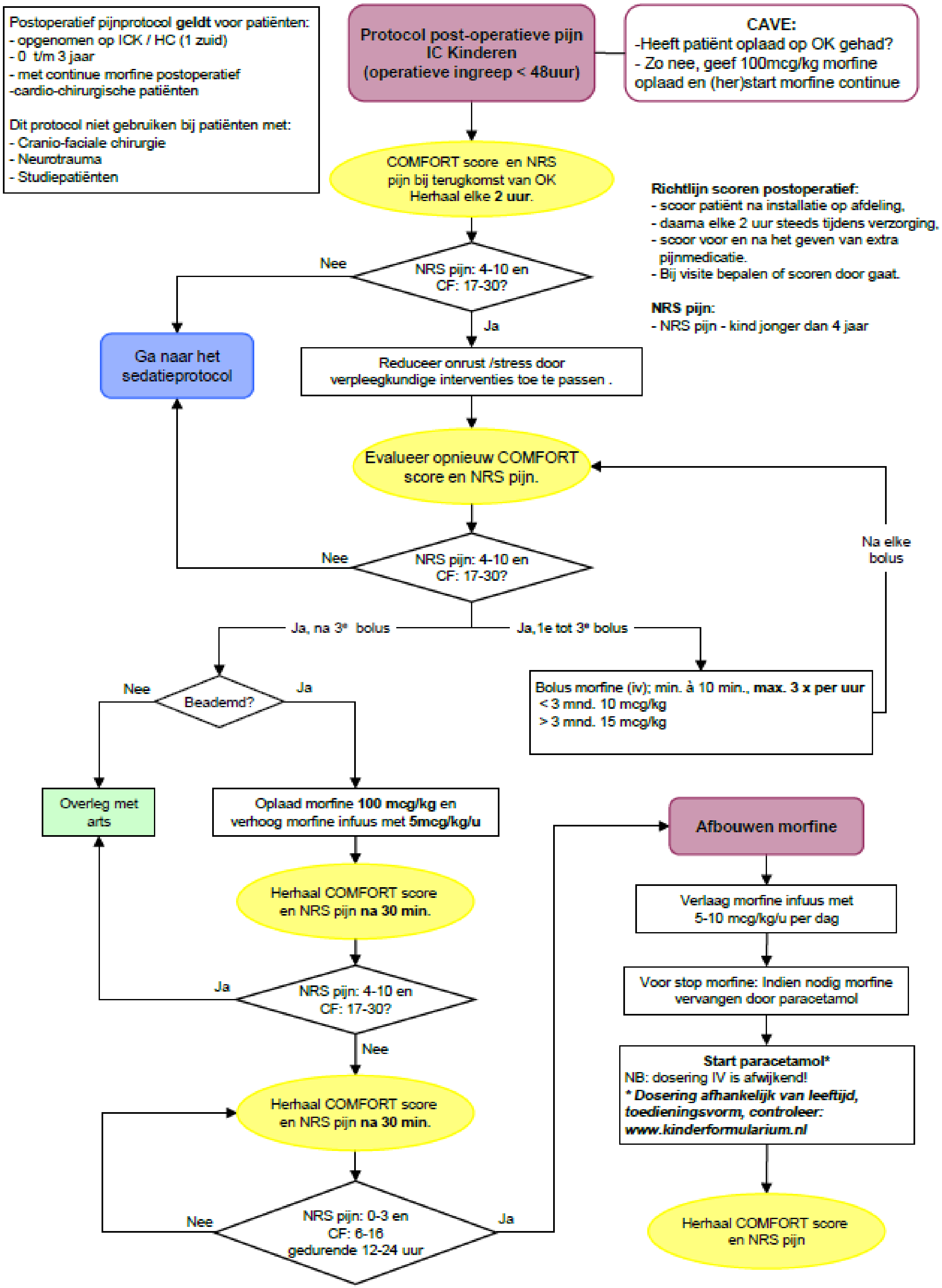

## Appendix 5: Comfort assessment scale.

### Pain assessment: Comfort-B and NRS-pain; sedation assessment Comfort-B and VISS.

**Comfort** assessment

# Gedrag schaal

| Datum/tijdstip 1 | Datum/tijdstip 2 | Sticker met naam van patient |
|---|---|---|
| Datum/tijdstip 3 | Datum/tijdstip 4 | |

Kruis het juiste antwoord aan

| | | 1 | 2 | 3 | 4 | |
|---|---|---|---|---|---|---|
| **Alertheid** | 1. | ☐ | ☐ | ☐ | ☐ | Diep in slaap (ogen dicht, geen reactie op omgeving) |
| | 2. | ☐ | ☐ | ☐ | ☐ | Licht in slaap (ogen grotendeels gesloten, af en toe reactie) |
| | 3. | ☐ | ☐ | ☐ | ☐ | Slaperig (kind sluit vaak zijn ogen, reageert minder op omgeving) |
| | 4. | ☐ | ☐ | ☐ | ☐ | Wakker en alert (kind reageert op omgeving) |
| | 5. | ☐ | ☐ | ☐ | ☐ | Wakker en hyper-alert (overdreven reactie op veranderingen) |
| **Kalmte/agitatie** | 1. | ☐ | ☐ | ☐ | ☐ | Kalm (kind lijkt helder en rustig) |
| | 2. | ☐ | ☐ | ☐ | ☐ | Licht angstig (kind toont lichte onrust) |
| | 3. | ☐ | ☐ | ☐ | ☐ | Angstig (kind lijkt onrustig maar kan zich beheersen) |
| | 4. | ☐ | ☐ | ☐ | ☐ | Zeer angstig (kind lijkt zeer onrustig, kan zich nog net beheersen) |
| | 5. | ☐ | ☐ | ☐ | ☐ | Paniekerig (ernstige onrust met verlies van beheersing) |
| **Ademhalingsreactie** (scoren bij beademde kinderen) | 1. | ☐ | ☐ | ☐ | ☐ | Geen hoesten en geen spontane ademhaling |
| | 2. | ☐ | ☐ | ☐ | ☐ | Spontane ademhaling met weinig of geen reactie op de beademing |
| | 3. | ☐ | ☐ | ☐ | ☐ | Af en toe hoesten of verzet tegen de ventilator |
| | 4. | ☐ | ☐ | ☐ | ☐ | Ademt actief tegen de ventilator in of hoest regelmatig |
| | 5. | ☐ | ☐ | ☐ | ☐ | Vecht tegen de ventilator; hoesten, verslikken, tegenademen |
| **Huilen** (scoren bij niet beademde kinderen) | 1. | ☐ | ☐ | ☐ | ☐ | Geen huilgeluiden |
| | 2. | ☐ | ☐ | ☐ | ☐ | Af en toe snikken of kreunen (nasnikken) |
| | 3. | ☐ | ☐ | ☐ | ☐ | Jengelen of dreinen (monotoon geluid) |
| | 4. | ☐ | ☐ | ☐ | ☐ | Huilen |
| | 5. | ☐ | ☐ | ☐ | ☐ | Schreeuwen of krijsen |
| **Lichaamsbeweging** | 1. | ☐ | ☐ | ☐ | ☐ | Geen beweging |
| | 2. | ☐ | ☐ | ☐ | ☐ | Incidentele (3 of minder) kleine bewegingen |
| | 3. | ☐ | ☐ | ☐ | ☐ | Frequente (meer dan 3) kleine bewegingen |
| | 4. | ☐ | ☐ | ☐ | ☐ | Heftige bewegingen met armen en benen |
| | 5. | ☐ | ☐ | ☐ | ☐ | Heftige bewegingen ook met romp en hoofd |
| **Spierspanning** | 1. | ☐ | ☐ | ☐ | ☐ | Spieren volledig ontspannen; geen spierspanning |
| | 2. | ☐ | ☐ | ☐ | ☐ | Verminderde spierspanning; minder weerstand dan normaal |
| | 3. | ☐ | ☐ | ☐ | ☐ | Normale spierspanning |
| | 4. | ☐ | ☐ | ☐ | ☐ | Toegenomen spierspanning en buiging van vingers en tenen |
| | 5. | ☐ | ☐ | ☐ | ☐ | Extreme spierstijfheid en buiging van vingers en tenen |
| **Gelaatsspanning** | 1. | ☐ | ☐ | ☐ | ☐ | Gezichtsspieren volkomen ontspannen |
| | 2. | ☐ | ☐ | ☐ | ☐ | Normale spanning van het gelaat |
| | 3. | ☐ | ☐ | ☐ | ☐ | Spanning duidelijk in sommige gelaatsspieren (niet aanhoudend) |
| | 4. | ☐ | ☐ | ☐ | ☐ | Spanning duidelijk in alle gelaatsspieren (aanhoudend) |
| | 5. | ☐ | ☐ | ☐ | ☐ | Gelaatsspieren verwrongen en in een grimas |
| **Total score** | | ☐ | ☐ | ☐ | ☐ | |
| **NRS pijn*** | | .... | .... | .... | .... | (0=geen pijn tot 10=ergste pijn) |
| **VISS*** | | .... | .... | .... | .... | Vul in: 1. onvoldoende sedatie, 2. adequate sedatie or 3. te diepe sedatie |

**Details sedativa/analgetica** ______________________________

**Reden meting** ______________________________

*Afkortingen: NRS = Numerieke Rating Schaal, VISS = Verpleegkundige Interpretatie van Sedatie Schaal

© Copyright Nederlandse versie HM Koot, JB de Boer en M van Dijk, versie 3, oktober 2002. Niet reproduceren zonder toestemming

## Appendix 6: Numeric rating Scale

**Example of visual analogue scale**

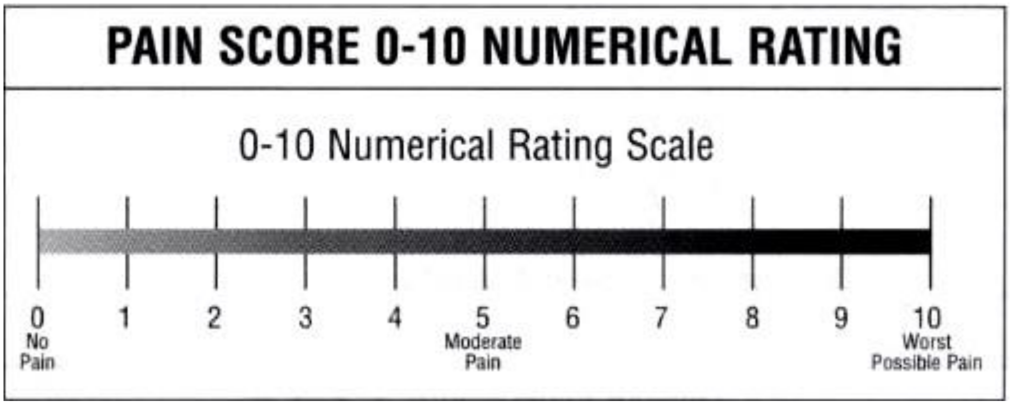

## Appendix 7: Parents Postoperative Pain Measure-Short Form

**Telephone interviews will take places two days after discharge from the hospital. The validated PPPM-SF will be used (95).**

**Table 2.** Instructions And Response Options for the PPPM-SF

Parents' Postoperative Pain Measure – Short Form (PPPM-SF).
Children sometimes have changes in behavior when recovering from surgery. The following is a list of behaviors that your child may or may not have exhibited while recovering from surgery between ______and______ today. For each of the behaviors below, circle the appropriate response, yes or no.
When your child was recovering from surgery between ______ and ______ today, did s/he…

| Long Form Item Number* | | | Circle One | |
|---|---|---|---|---|
| 1 | 1 | Whine or complain more than usual? | Yes | No |
| 3 | 2 | Play less than usual? | Yes | No |
| 4 | 3 | Not do the things s/he normally does? | Yes | No |
| 5 | 4 | Act more worried than usual? | Yes | No |
| 6 | 5 | Act more quiet than usual? | Yes | No |
| 7 | 6 | Have less energy than usual? | Yes | No |
| 9 | 7 | Eat less than usual? | Yes | No |
| 10 | 8 | Hold the sore part of his/her body? | Yes | No |
| 12 | 9 | Groan or moan more than usual? | Yes | No |
| 14 | 10 | Want to be close to you more than usual? | Yes | No |

Note on Administration and Scoring: Parents are asked to complete the measure between a specific time period (ie, between breakfast and lunch, between lunch and supper, or supper and bedtime). The number of items parents have circled "Yes" are summed for a total score out of 10. A score of at least 3 out of 10 signifies clinically significant pain.
*Long form item numbers are given for reference only and should be omitted in administering the PPPM-SF.

Kinderen gedragen zich soms anders als ze herstellen na een operatie. Op de onderstaande lijst staat bepaald gedrag dat uw kind wel of niet heeft laten zien na de operatie. Geef voor elke punt aan of dit van toepassing is door ja of nee te antwoorden.

De volgende vragen gaan over het gedrag van uw kind na het ontslag uit het ziekenhuis.

- Zeurt of klaagt uw kind meer dan normaal? Ja/nee
- Speelt uw kind minder? Ja/nee
- Is uw kind niet bezig met wat hij of zij normaal doet? Ja/nee
- Is uw kind meer ongerust dan normaal? Ja/nee
- Is uw kind stiller dan normaal? Ja/nee
- Heeft uw kind minder energie dan normaal? Ja/nee
- Eet uw kind minder dan normaal? Ja/nee
- Raakt uw kind de pijnlijke plek (van het lichaam) steeds aan? Ja/nee
- Kreunt en kermt uw kind meer dan normaal? Ja/nee
- Is uw kind aanhankelijker dan normaal? Ja/nee

Vertaalde versie van de PPPM-SF ontwikkeld door von Baeyer et al. (95)

Comfort assessment

# SOS-PD schaal

Sophia Ontwenningsverschijnselen Scorelijst-Pediatrisch Delirium

| Datum/tijd 1 | Datum/tijd 2 | |
|---|---|---|
| | | Sticker met naam van patiënt |
| Beoordelaar | Beoordelaar | |
| | | |

| Step 1a Ontwenning | 1 | 2 | Toelichting |
|---|---|---|---|
| Hartfrequentie | ..... /min | ..... /min | Noteer hoogste waarde in de afgelopen 4 uur indien beschikbaar (elektronisch 'patient data management system'), anders huidige waarde aflezen van de monitor of pols voelen. |
| Ademhalingsfrequentie | ..... /min | ..... /min | Noteer hoogste waarde in de afgelopen 4 uur indien beschikbaar (elektronisch patient data management system'), anders huidige waarde aflezen van de monitor of ademhaling tellen. |
| Baseline hartfrequentie | ..... /min | ..... /min | z.o.z. voor toelichting |
| Baseline ademhalings-frequentie | ..... /min | ..... /min | z.o.z. voor toelichting |

| Stap 1b Delirium* | 1 | 2 | Kruis aan als symptoom aanwezig is |
|---|---|---|---|
| Ouders herkennen gedrag kind niet | ☐* | ☐* | Ouders beleven het gedrag van hun kind als zeer afwijkend of onherkenbaar dan wat zij gewend zijn bij ziekte of ziekenhuisopname, "dit is mijn kind niet." |

| Stap 2 | Ontwenning 1 | Ontwenning 2 | Delirium 1 | Delirium 2 | |
|---|---|---|---|---|---|
| Tachycardie | ☐ | ☐ | | | Hartfrequentie 15% of meer boven de baselinewaarde. |
| Tachypnoe | ☐ | ☐ | | | Ademhalingsfrequentie 15% of meer boven de baselinewaarde. |
| Koorts | ☐ | ☐ | | | Lichaamstemperatuur > 38.4 °C nu of in afgelopen 4 uur . |
| Zweten | ☐ | ☐ | ☐ | ☐ | Zonder aanwijsbare reden |
| Agitatie / rusteloosheid | ☐ | ☐ | ☐ | ☐ | Bijvoorbeeld: prikkelbaar, opgewonden, rusteloos, plukkerig (trekt lijn, infuus, katheters eruit of doet pogingen). |
| Angst | ☐ | ☐ | ☐ | ☐ | Bijvoorbeeld: ogen wijd open, wenkbrauwen aangespannen en omhoog getrokken. |
| Tremoren | ☐ | ☐ | ☐ | ☐ | Kleine onwillekeurige ritmische bevingen van bijv. handen of voeten (soms in reactie op prikkels, bijv. verzorging, omgevingsgeluiden). |
| Motorische onrust | ☐ | ☐ | ☐ | ☐ | Onwillekeurige bewegingen van armen en/of benen |
| Toegenomen spierspanning | ☐ | ☐ | ☐ | ☐ | Bijvoorbeeld: gebalde vuisten, of opgetrokken schouders OF: afwijkende gespannen houding van hoofd, armen en/of benen. |
| Verminderde aandacht | | | ☐ | ☐ | Ouders beleven het gedrag van hun kind als zeer afwijkend of onherkenbaar dan wat zij gewend zijn bij ziekte of ziekenhuisopname, "dit is mijn kind niet." |
| Handelt niet doelgericht | | | ☐ | ☐ | Het kind heeft moeite heeft met handelingen die normaal geen probleem zijn. Bijvoorbeeld: het lukt niet goed om speen of knuffel te pakken |
| Oogcontact verminderd / afwezig | | | ☐ | ☐ | Het kind heeft geen of verminderd oogcontact met verpleegkundige of ouders. |
| Ontroostbaar huilen | ☐ | ☐ | ☐ | ☐ | Als huilen niet stopt door troosten met bijvoorbeeld speen of voeding; of bij oudere kinderen door aanbieden van spel. Bij geïntubeerde kinderen geluidloos huilen als ja scoren. |
| Grimassen | ☐ | ☐ | ☐ | ☐ | Gefronste wenkbrauwen, zichtbare neus-mondplooi, samen- of dichtgeknepen ogen. |
| Slapeloosheid / verstoord patroon | ☐ | ☐ | ☐ | ☐ | Als het kind niet langer dan 1 uur aaneengesloten slaapt; hazenslaapjes. |
| Hallucinaties* | ☐ | ☐ | ☐ | ☐* | Als het lijkt alsof het kind in de afgelopen 4 uur dingen zag, hoorde of voelde die er niet waren. |
| Desoriëntatie (tijd/ plaats/persoon) | | | ☐ | ☐ | Alleen voor bij kinderen > 5 jr. Als het kind niet weet of het ochtend, middag of avond is, waar het is, ouders en bekenden niet herkent. |
| Verstoorde spraak | | | ☐ | ☐ | Kind praat onverstaanbaar, verhaal is niet te volgen (niet leeftijd-adequaat). |
| Braken | ☐ | ☐ | | | Minstens eenmaal tijdens de afgelopen 4 uur. |
| Diarree | ☐ | ☐ | | | Minstens eenmaal tijdens de afgelopen 4 uur. |
| Acuut begin symptomen | | | ☐ | ☐ | Acute verandering van symptomen t.o.v. voor de opname |
| Fluctuerend verloop | | | ☐ | ☐ | De aanwezigheid van symptomen wisselt sterk over de afgelopen 24 uur. |

Total score

| | | | |
|---|---|---|---|
| SOS-score | ☐ ☐ | | Ontwennings-score (maximum 15) tel aantal aangekruiste vakjes |
| PD-score* | | ☐ ☐ | Delirium-score (maximum 16/17) tel aantal aangekruiste vakjes |

*** Overleg kinder- en jeugdpsychiater als: Stap 1b is positief EN/ OF Stap 2 score is ≥4 of symptoom met * is positief.**

Z.o.z. voor instructies.

©Copyright Dutch and English version E. Ista, M. van Dijk, M. de Hoog - version 2.0 October 2014. Do not reproduce without permission.

## Appendix 9: anaesthetic management

Pre-medication (midazolam) at the discretion of the anaesthesiologists

Induction of anaesthesia*

Connecting monitoring devices **

Start of surgery

Start of CPB

Weaning of CPB

First PK sample (see sampling schedule in protocol)

Loading dose morphine 100 mcg/kg

End of surgery

Transition to the ICU

Start of study medication, (IV morphine or IV paracetamol and placebo)

All peri-operative drugs are registered at the electronic data system. Also, all information on the perfusion, i.e. CPB run time, degree and duration of hypothermia, are registered. Information that is suspected to influence the PK will be added to the database.

* Induction of anaesthesia will be achieved by sevoflurane 8%, midazolam 0.1-0.3 mg/kg, sufentanil 1-3 mcg/kg and pancuronium 0.1-0.2 mg/kg. Per-operative anesthesia is maintained by continuous midazolam infusion and sufentanil shots if necessary. Several other standardized drugs are used, such as antibiotics.

** Monitoring devices will be connected before and after induction of anaesthesia, depending on the type of monitoring. Monitoring consists of ECG, invasive blood pressure, central venous pressure, oxy-haemoglobin saturation, end-tidal carbon dioxide levels, temperature (cerebral and central), bispectral index (BIS), brain tissue oxygen saturation (INVOS), transoesofageal ultrasound, urine catheter. NIRS sensors will also be used as standard post-operative monitoring of cerebral oxygenation.

**Appendix 10: monitoring plan**

## Frequentie en Inhoud Monitoring voor ONDERZOEK MET HOOG RISICO

## FREQUENTIE MONITORING

3 of meer visite per jaar per centrum afhankelijk van inclusiesnelheid en eerder geobserveerde deviaties.

## INHOUD MONITORING

**Studiedocumenten en afspraken**

- Controle aanwezigheid en volledigheid van het onderzoeksdossier: Trial Master File en Investigator File.
- Controle instructies studiepersoneel en afspraken over back-up door bevoegde collega's.

**Patiënteninstroom, consent, compliance en Source Document Verification (SDV)**

- Controle inclusiesnelheid en uitval percentage.
- Controle informed consent: steekproef: 25%.
- Controle in- en exclusiecriteria: steekproef: eerste 3 deelnemers, daarna 50-100%.
- Controle protocolcompliance: steekproef: eerste 3 deelnemers, daarna 50-100%.
- Source Document Verification (SDV) steekproef: 50-100%. Wordt uitgevoerd op basis van een tevoren gedefinieerde lijst van variabelen - inclusief primair eindpunt - die in duidelijke relatie staan tot de veiligheid en geldigheid van het onderzoek.

**Patiëntveiligheid**

- Controle Serious Adverse Event (SAE) reporting: steekproef 50-100 % van de proefpersonen.
- Controle Suspect Unexpected Serious Adverse Reaction (SUSAR) reporting: steekproef 50-100 % van de proefpersonen (indien van toepassing).

**Studiemedicatie of onderzoeksproduct (indien van toepassing)**

- Controle instructies deelnemers.
- Controle overzichten van ontvangst, opslag, uitgifte, retour en voorraadbeheer.
- Controleer aanwezigheid correcte procedure voor deblindering in geval van nood.

**Studieprocedures**

- Controle of instructies voor uitvoer van studieprocedures aanwezig zijn.
- Controle apparatuur en /of faciliteiten.

**Laboratorium, apotheek en biologische monsters (indien van toepassing)**

- Controle of laboratoria GLP gecertificeerd zijn.
- Controle of apotheken GMP gecertificeerd zijn.
- Controle verzameling, labeling en opslag van biologische monsters.

## AANDACHTSPUNTEN

- kwalificaties monitor
- terugkoppeling en follow-up van bevindingen van de monitor
    - Termijn van beschikbaarheid monitoring-rapporten.
    - Acties naar aanleiding van verbeterpunten in monitoring rapport: binnen Erasmus MC en in andere deelnemende centra (in geval van multcenter trial).
- bewaren van studiegegevens
    - Gebruik van een adequaat Clinical Data Management Systeem (CDMS).
    - Correct bewaren van ruwe gegevens, gecorrigeerde gegevens en back-ups.
    - Beschikbaarheid van een audit trail (herleidbaarheid van studiedata en aanpassingen).

## MONITORINGRAPPORTEN EN BEWAARTERMIJN

Van elke monitoringvisit wordt een schriftelijk verslag gemaakt, het monitoringrapport. Het afdelingshoofd van de hoofdonderzoeker is verantwoordelijk voor de archivering van de monitoringrapporten gedurende minimaal 15 jaar na afronding van het onderzoek. De monitoringrapporten en overige onderzoeksdocumenten zijn op verzoek toegankelijk voor de Raad van Bestuur van het Erasmus MC, en voor door de Raad van Bestuur geautoriseerde medewerkers.

*Bijlage C "Hoog Risico" bij het Erasmus MC Monitoringplan - versie 19 februari 2013. Deze bijlage is niet geldig als apart document*

*Deze bijlage is enkel te gebruiken als onderdeel van het door de hoofdonderzoeker ondertekende Erasmus MC monitoringplan.*

# Monitoringplan

**WMO-plichtig onderzoek**

**Erasmus MC is verrichter**

## ALGEMENE GEGEVENS ONDERZOEK

| | |
|---|---|
| NL nummer (ABR) | NL53085.078.15 |
| MEC nummer | Volgt |
| Erasmus MC Afdeling | IC kinderen |
| **Hoofdonderzoeker Erasmus MC** | Dr. E.D. wildschut |
| Protocol code nummer / acroniem | PACS |
| Protocol titel: Pediatric Analgesia after Cardiac Surgery; Morphine IV versus paracetamol IV after cardiac surgery in neonates and infants. | |

## BEPALING RISICOCLASSIFICATIE

**Bepaal het risico voor de patiënt aan de hand van de NFU tabel:**

| **Mate van Schade / GROOTTE VAN KANS** | **Lichte Schade** | **Matige Schade** | **Ernstige Schade** |
|---|---|---|---|
| **KLEINE KANS** | Verwaarloosbaar risico | Verwaarloosbaar risico | Matig risico |
| **MATIGE KANS** | Verwaarloosbaar risico | Matig risico | Hoog risico |
| **GROTE KANS** | Matig risico | Hoog risico | Hoog risico |

**Resultaat risicoclassificatie patiënt:**

☐ verwaarloosbaar risico

☒ matig risico

☐ hoog risico

**Bepaal het risico voor de wetenschappelijke kwaliteit van de data:**

Denk hierbij bijvoorbeeld aan de volgende punten:

Is het protocol moeilijk uit te voeren, bijvoorbeeld omdat het in grote mate afwijkt van de reguliere diagnostiek en/of zorg?

Zijn er andere knelpunten er ten aanzien van de betrouwbaarheid van de data en de wetenschappelijke kwaliteit? bijvoorbeeld erg uitgebreid CRF, weinig proefpersonen per centrum, …

**Resultaat risicoclassificatie wetenschap:**

☐ verwaarloosbaar risico

☐ matig risico

☒ hoog risico

**Het risico van het onderzoek volgt uit bovenvermelde risico's**

Houd van de hierboven bepaalde risicoclassificaties de hoogste aan. Dit is de risicoclassificatie van uw onderzoek.

**Resultaat beoordeling risicoclassificatie onderzoek:**

☐ verwaarloosbaar risico

☐ matig risico

☒ hoog risico

**MONITORING FREQUENTIE EN INHOUD**

**Het vastgestelde risiconiveau impliceert dat monitoring van dit onderzoek minimaal de activiteiten omvat die zijn vermeld in de betreffende bijlage:**

Onderzoek met verwaarloosbaar risico -> zie bijlage A

Onderzoek met matig risico -> zie bijlage B

Onderzoek met hoog risico -> zie bijlage C

**Voeg de betreffende bijlage toe; deze bijlage maakt integraal onderdeel uit van dit monitoringplan.**

**ONDERTEKENING DOOR HOOFDONDERZOEKER:**

NAAM: dr. E.D. Wildschut_____________________________________

FUNCTIE: kinderarts-intensivist ___________________________________

HANDTEKENING: _____________________________

DATUM ONDERTEKENING: 16-12-2015_____________________
